# Recent advances in molecular mechanisms of skin wound healing and its treatments

**DOI:** 10.3389/fimmu.2024.1395479

**Published:** 2024-05-21

**Authors:** Abdullah Al Mamun, Chuxiao Shao, Peiwu Geng, Shuanghu Wang, Jian Xiao

**Affiliations:** ^1^ Central Laboratory of The Lishui Hospital of Wenzhou Medical University, Lishui People’s Hospital, Lishui, Zhejiang, China; ^2^ Molecular Pharmacology Research Center, School of Pharmaceutical Sciences, Wenzhou Medical University, Wenzhou, China; ^3^ Department of Wound Healing, The First Affiliated Hospital of Wenzhou Medical University, Wenzhou, China

**Keywords:** skin, skin wound healing, immune cell-wound healing, wound physical therapy, stem cell therapy

## Abstract

The skin, being a multifaceted organ, performs a pivotal function in the complicated wound-healing procedure, which encompasses the triggering of several cellular entities and signaling cascades. Aberrations in the typical healing process of wounds may result in atypical scar development and the establishment of a persistent condition, rendering patients more vulnerable to infections. Chronic burns and wounds have a detrimental effect on the overall quality of life of patients, resulting in higher levels of physical discomfort and socio-economic complexities. The occurrence and frequency of prolonged wounds are on the rise as a result of aging people, hence contributing to escalated expenditures within the healthcare system. The clinical evaluation and treatment of chronic wounds continue to pose challenges despite the advancement of different therapeutic approaches. This is mainly owing to the prolonged treatment duration and intricate processes involved in wound healing. Many conventional methods, such as the administration of growth factors, the use of wound dressings, and the application of skin grafts, are used to ease the process of wound healing across diverse wound types. Nevertheless, these therapeutic approaches may only be practical for some wounds, highlighting the need to advance alternative treatment modalities. Novel wound care technologies, such as nanotherapeutics, stem cell treatment, and 3D bioprinting, aim to improve therapeutic efficacy, prioritize skin regeneration, and minimize adverse effects. This review provides an updated overview of recent advancements in chronic wound healing and therapeutic management using innovative approaches.

## Graphical Abstract

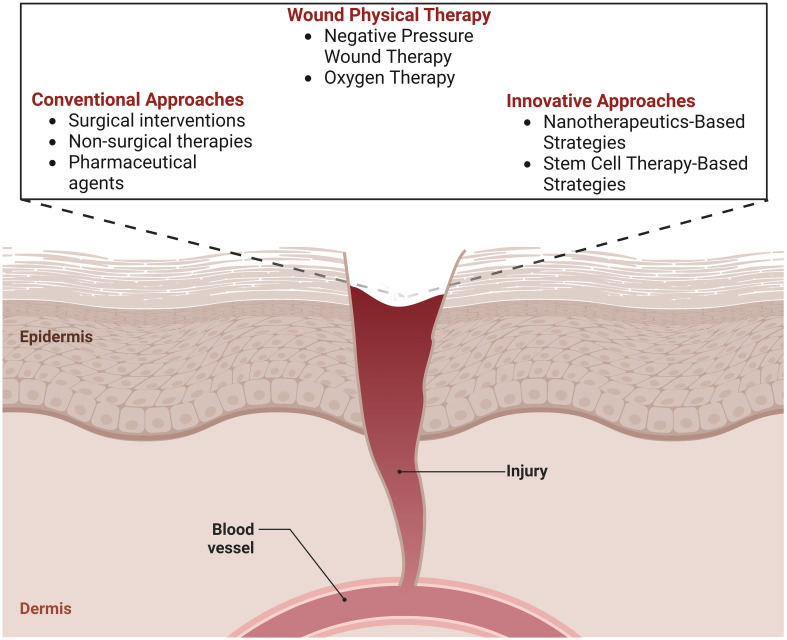

## 1 Introduction

Skin is the largest organ of our body, which accounts for approximately 16% of the total body weight. It is essential for both the maintenance of homeostasis and the functioning of the barrier it provides against the impact of external stimuli (1). Skin integrity is of utmost importance in the preservation of overall health, given that injuries resulting from chronic disorders, burns, trauma, and surgical procedures may lead to impairment and emotional anguish. These consequences provide a significant roadblock to healthcare systems worldwide (2). Multiple cell types must be synchronized sequentially for skin healing. The epidermis is impermeable in healthy skin and protects against external factors (3). The epidermis, which encompasses hair follicles, sebaceous, and sweat glands, provides structural integrity, immunity, and nutrition to the integumentary system. The dermis is characterized by higher levels of extracellular matrix (ECM), vascular, and mechanoreceptors, while subcutaneous adipose tissue functions as an energy reservoir and a consistent supply of growth factors (4). Each skin layer has immune system cells that monitor damage to the skin. Several kinds of cells in various layers must cooperate at crucial stages to repair skin wounds (5, 6). Based on causes and consequences, wounds may be healing (acute) and non-healing (chronic). The healing process of minor acute wounds is mainly facilitated by the inherent regenerative capacity of the skin, which involves cellular mechanisms, remodeling of the ECM, and the presence of growth factors (5, 7). Injuries to the skin and chronic wounds heal slowly owing to infection and fluid loss. These injuries damage the skin’s structure, harming millions and presenting economic and societal problems to global healthcare systems (8, 9). Despite the considerable amount of research conducted, there are still unmet needs in the field of skin wound healing techniques and the treatment of extensive and chronic wounds. These issues primarily arise from difficulties in accurately assessing wounds and effectively managing their care. Hence, the development of upgraded and novel approaches for healing skin wounds has substantial medical significance worldwide (3, 8, 10).

Skin healing from wounds is paramount for sustaining the quality of life and reaching the goal of wound closure. It encompasses a multitude of cell types and mediators operating in a highly intricate sequence (11). Despite the existence of a substantial body of research on the processes behind wound healing, there are several unresolved issues regarding its physiologic regulation (12, 13). Chronic wounds are now a growing problem, being identified at a concerning rate and imposing a substantial economic strain on the medical field. The increasing prevalence of chronic wounds may be attributed to aging populations and the rising rates of obesity and diabetes on a global scale. Consequently, this has resulted in a substantial escalation in the economic costs associated with the management and treatment of these persistent wounds (9). The yearly expenditure to treat chronic wounds exceeds $25 billion, a substantial amount that becomes even more significant when accounting for the additional expenses associated with decreased productivity among afflicted persons and the provision of long-term care in facilities and nursing homes (14). Diabetic foot ulcers and pressure ulcers are significant contributors to morbidity rates and impose a substantial economic burden (15). The investigation of tissue regeneration in chronic wound healing is of utmost importance due to the rising occurrence of diabetes and obesity and the demand for wound care among veterans. The investigation of tissue regeneration in the context of chronic wound healing is crucial in order to effectively tackle these challenges and enhance the overall well-being of patients (9).

Skin wound therapy may be categorized into two main groups: conventional procedures and regenerative approaches. Traditional methods of treating wounds often include measures to limit infection, regular changes of dressings, and the removal of wrecked tissues by debridement (16). Split-thickness skin autografts are a vital medical intervention with limitations like repeated surgeries, limited donor sites, hypertrophic damage, and functional changes. Regenerative wound healing encompasses a range of developing biomedical technologies, such as bioactive biomaterials, innovative dressings for wounds, treatment with stem cells, growth factor administration, gene therapies, and bioengineered skin grafts. These innovative approaches attempt to restore the skin’s original function and repair damaged tissues. This technique enables faster and higher-quality wound healing while minimizing the occurrence of scarring (17, 18). A previous wound healing method centered on layer-by-layer skin regeneration utilizing bioengineered scaffolds or cell-encapsulated hydrogels. Rapid wound healing with little scarring has been achieved using cell-laden matrices, including fibroblasts, keratinocytes, or stem cells (19). Nevertheless, there is potential for enhancement in the spatial distribution and cellular composition to replicate the intricate microarchitecture seen in natural skin tissues (20). Hence, the current emphasis is primarily on the advancement of diverse, developing, and creative approaches to expedite the wound healing process while ensuring the preservation of functional attributes. Within emerging treatment modalities, there has been significant interest in using stem cell-based therapies, specifically those employing various types of stem cells, such as induced pluripotent stem cells and mesenchymal stem cells. These therapies are being explored extensively in clinical and preclinical studies as a component of stem cell-based regenerative medicine (21). The efficacy of stem cell-based therapy is limited by low wound stem cell survival after implantation/grafting owing to a hostile inflammatory environment (22). Stem cell viability enhancement, genome editing, and genetic alterations offer promising methods for personalized wound care for patients with enduring wounds (23).

The main objective of wound treatment is to enhance the accuracy of diagnosis and prognosis to develop individualized treatment strategies. Over the past decade, there has been a significant emphasis on exploring innovative therapeutic methods, such as nanotherapeutics, stem cell therapy, and 3D bioprinting (24, 25). The present article presents an in-depth review of recent breakthroughs in treatments for wound healing. It delves into the molecular mechanisms behind this process, explores traditional treatment modalities, and highlights newer tactics that show promise in this field. The review encompasses an examination of nanotherapeutic methodologies, including the use of nanomaterials, and an exploration of the application of stem cell treatment. Additionally, the article addresses the many difficulties encountered in contemporary wound healing therapy and explores potential advancements in this field.

## 2 The molecular mechanisms of healing process

**Figure 1:**
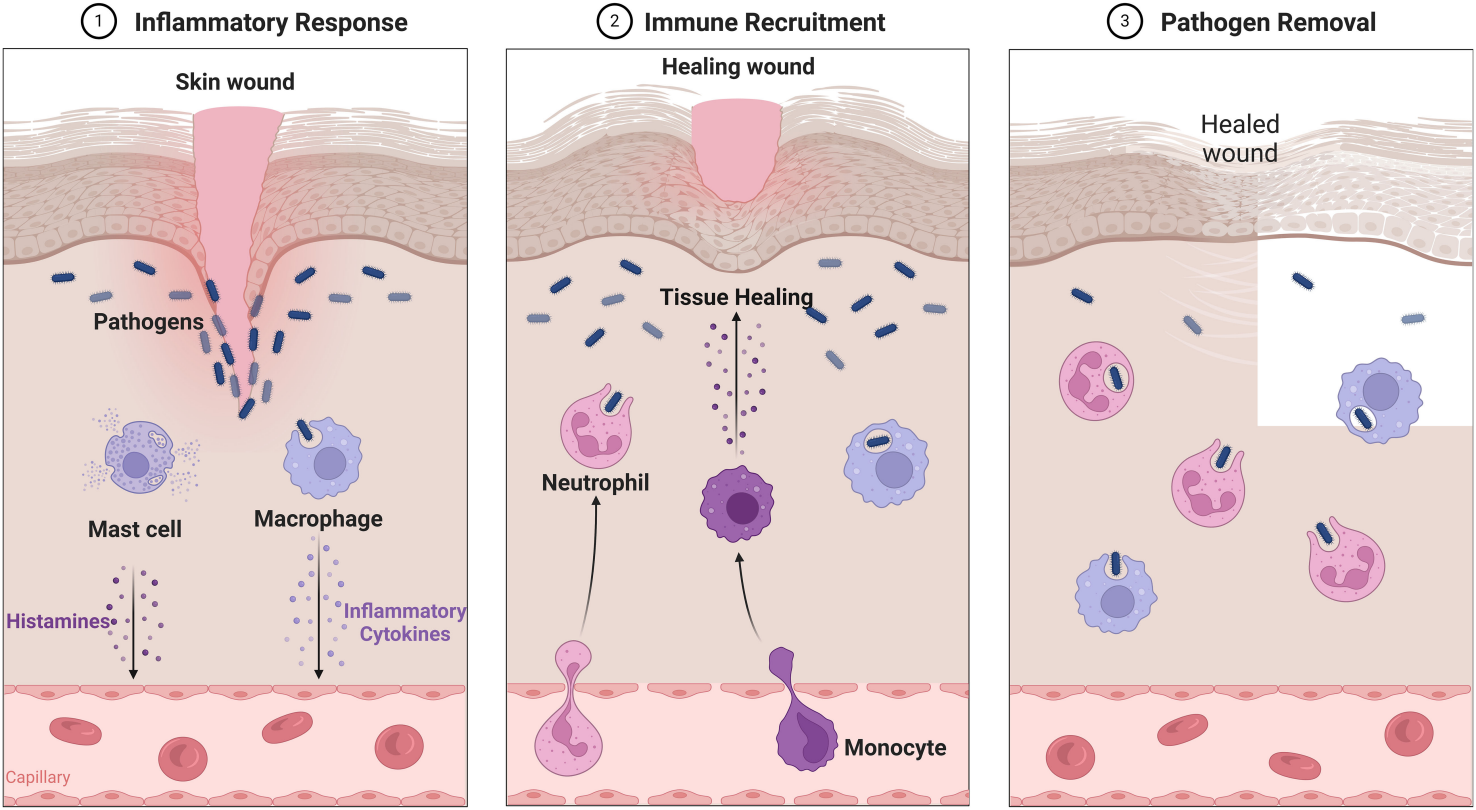
Overview of Wound healing.

Skin healing from wounds is a complex phenomenon that incorporates several cellular, humoral, and molecular pathways. It starts promptly upon the appearance of a lesion and may persist for an extended duration, perhaps spanning several years (21). The condition under consideration comprises instances of tissue disruptions resulting in functional impairment. Intrinsic injuries may manifest as either open wounds, which occur on the surface of the body or closed wounds, which entail ruptures to internal organs while the skin remains intact. Either regeneration or repair mechanisms may attain closure. Regeneration entails the replacement of damaged tissue, while skin healing includes the process of fibrosis and subsequent scar formation (26). The phenomenon is sometimes characterized as a symphony or theatrical performance, whereby the interaction among cells, growth factors, and cytokines culminates in the closing of the skin (27). Nevertheless, current research indicates that the insufficiency of a particular cell type or the lack of a mediator may be compensated for by other entities participating in the wound healing process (**Figure 1**), thus enabling the progression of repair (**Table 1**) (30, 34).

**Table 1:** The role and functions of different types of cells in skin wound healing.

Cell Type	Role and Functions	Ref.
**Keratinocytes**	- Primary cells of the epidermis, responsible for forming the outer layer of the skin (epidermal barrier)	(28)
	- Proliferate and migrate to cover the wound surface (re-epithelialization)	
	- Secrete growth factors and cytokines to promote healing	
**Fibroblasts**	- Produce collagen, elastin, and other extracellular matrix components necessary for tissue repair	(29)
	- Responsible for wound contraction, reducing the wound size	
**Inflammatory Cells**	- Neutrophils: First responders to injury, involved in clearing debris and pathogens through phagocytosis	(30)
	- Macrophages: Clean up cellular debris, release growth factors to promote tissue repair, and regulate inflammation	
	- Mast Cells: Release histamine and other inflammatory mediators to initiate the inflammatory response	
**Endothelial Cells**	- Form new blood vessels (angiogenesis) to restore blood supply to the wound area	(31)
	- Provide a scaffold for migrating cells during wound healing	
	- Secrete growth factors to stimulate other cells involved in wound repair	
**Platelets**	- Form blood clots (hemostasis) to stop bleeding	(32)
	- Release growth factors such as platelet-derived growth factor (PDGF) and transforming growth factor-beta (TGF-β) to stimulate tissue repair	
	- Attract other cells involved in wound healing	
**Mesenchymal Stem Cells**	- Can differentiate into various cell types involved in wound healing, including fibroblasts, endothelial cells, and keratinocytes	(33)
	- Secrete factors that modulate inflammation and promote tissue regeneration	
	- Enhance angiogenesis and collagen deposition	

The efficient process of healing a wound, free from any adverse outcomes, is of utmost importance for the life of an individual. It reinstates the structural integrity of the skin and serves as a protective barrier against potential risks such as dehydration and infection (35). The process of wound healing in adults encompasses a sequence of processes that culminate in the restoration of damaged tissues and the development of scar tissue. The healing process is comprised of several stages of proliferation, inflammation, hemostasis, and remodeling, which occur in a coordinated manner (36). The number of inflammatory macrophages and monocytes infiltrates the tissue more deeply as time goes on, eventually reaching a peak during the proliferative period before gradually decreasing. Early in the process, lymphocytes in the blood migrate to the skin, achieving a point of saturation by day four and continuing to do so for the next two weeks before beginning to decrease. The third step, which involves redesigning the tissue and forming a scar to reinstate the skin’s integrity, occurs in the second week following the damage. This procedure can take many months to complete. This review offers up-to-date information on the critical role that the microenvironment, immune cells, and the connections between these factors play in the process of wound healing (37–39).

### 2.1 Hemostasis

Acute wounds are characterized by vascular damage and hemorrhaging, and the first stage of wound healing involves the implementation of measures to mitigate blood loss by means of vasoconstriction and the production of blood clots. The process of hemostasis is triggered when blood components come into contact with the inner lining of blood vessels, leading to the adhesion, aggregation, and formation of an initial hemostatic plug by platelets (35). The procedure mentioned above unfolds via three distinct stages: vasoconstriction, primary hemostasis, and secondary hemostasis. The platelet, which serves as the crucial cell, interacts with fibrinogen, a vital matrix component. The healthy endothelilal cells (ECs) monolayer protects platelets in undamaged skin, preventing premature activation (26). Fibrinogen, which is synthesized by hepatocytes, is distributed throughout the bloodstream and is found inside platelets; nevertheless, it remains unprocessed into fibrin fibers, which are crucial constituents of blood clot formation (40, 41).

In the case of skin injury, the blood arteries inside the skin undergo constriction to impede hemorrhaging. As mentioned above, the process is characterized by the sequential occurrence of primary and secondary hemostasis, which are mediated by two interrelated routes (42). Primary hemostasis encompasses the processes of aggregation of platelets and the development of a platelet plug, which are initiated by the exposure of collagen in the sub-endothelial matrix. Secondary hemostasis is the process by which the coagulation cascade is triggered, leading to the conversion of soluble fibrinogen into insoluble strands that ultimately form a fibrin mesh. The formation of a thrombus serves to halt hemorrhaging while also facilitating the release of complements and growth factors, promoting wound healing via the provision of a supportive scaffold (43).

#### 2.1.1 Vasoconstriction

Following an injury, blood arteries undergo a fast constriction process to minimize bleeding from damaged microvasculature. The method of achieving this phenomenon involves the reflexive contraction of vascular smooth muscle, which is initiated by vasoconstrictors such as endothelin (44). The regulation of vasoconstriction is also influenced by circulating catecholamines, such as epinephrine, norepinephrine, and prostaglandins. Platelets are responsible for the production of platelet-derived growth factor (PDGF). This signaling molecule stimulates the activation of mesenchymal cells, namely smooth muscle cells located inside the walls of blood vessels (45).

Nevertheless, the first reflexive contraction of muscles only temporarily reduces bleeding. This is due to the fact that the wound experiences a rise in hypoxia and acidity, leading to passive muscle relaxation and subsequent resumption of bleeding. The subsequent activation of the coagulation cascade is necessary to control the process of vasoconstriction further and facilitate the resolution of long-term bleeding. Vasoconstriction, accompanied by clot formation, is crucial in preserving life by preventing excessive blood loss. However, this physiological response may also result in impaired local perfusion, heightened glycolytic activity, and alterations in pH levels (43, 46).

#### 2.1.2 Formation of the platelet plug

After an injury and the subsequent rupture of blood vessels, the thrombogenic subendothelial matrix becomes exposed, which facilitates the binding of platelets and initiates the inside-out signaling route for platelet activation. Consequently, this phenomenon results in the activation of integrins, promoting enhanced platelet adhesion to both neighboring platelets and the adjacent ECM. The external-to-internal signaling route enhances platelet activation and regulates the actin cytoskeleton, resulting in the transformation of the platelet into a cell with a fried-egg-like morphology that exhibits robust adhesion to the ECM, undergoes contraction, and effectively occludes the blood a blood vessel (39, 47, 48). The surface area of the activated platelet is increased as a result of the fusion between intracellular granules and the plasma membrane or surface-connected membranes of the open canalicular system (OCS). The granules mentioned above are responsible for the secretion of more than 300 bioactive compounds, including ADP, serotonin, calcium, histamine, as well as vWF and integrins, which play crucial roles in both primary and secondary hemostasis (42, 49). The surface area of the activated platelet is increased as a result of the fusion between intracellular granules and the plasma membrane or surface-connected membranes of the OCS. The granules, as mentioned above, are responsible for the secretion of more than 300 bioactive compounds, including ADP, serotonin, calcium, histamine, as well as vWF and integrins, which play crucial roles in both primary and secondary hemostasis (50). Activated platelets also secrete molecules such as thromboxane A2, which enhance platelet aggregation, resulting in the formation of the “platelet plug.” The release of cytokines and growth factors by platelets inside the plug plays a crucial role in cellular mediation for the process of healing. The intensity of platelet factor release is highest during the first hour after activation. However, the release of these factors by activated platelets persists for seven days, hence imposing paracrine impacts on many cell types present in the wound (47).

#### 2.1.3 Coagulation and reinforcement of the platelet plug

Platelets are crucial in assembling and activating coagulation complexes, triggered by contact with the subendothelial matrix, with traditional coagulation routes recognized in the field. These pathways are triggered upon contact with the subendothelial matrix. The activation of Factor X initiates the process of fibrin synthesis, ultimately resulting in the development of a blood clot. The blood clot consists of cross-linked fibrin, along with cellular components such as erythrocytes and platelets. Additionally, various ECM proteins, including fibronectin, vitronectin, and thrombospondin, are present in the clot (6, 51). Factor XIII is responsible for the covalent crosslinking of fibrin, which results in the binding of the aggregated platelet plug. This process leads to the formation of a final secondary hemostasis plug, also known as the thrombus. The thrombus functions as the temporary ECM for the invasion of various cells throughout the following phases of the healing process. The adhesion of platelets is facilitated by activated integrin receptors located on their surface (52, 53). The adhesion of platelets is facilitated by activated integrin receptors located on their surface. Platelets in the clot degranulated, producing powerful inflammatory cell chemoattractants, local fibroblast and EC activation factors, and vasoconstrictors. CCL5, thrombin, TGF β, PDGF, and VEGF are important chemokines that regulate coagulation and restrict blood vessel formation (54). The process of fibrin breakdown and the subsequent activation of the complement system are essential components in initiating the inflammatory response, promoting the formation of new blood vessels in wounds, and enabling the proliferation of stromal cells. Fibrin interacts with integrin CD11b/CD18 on invading monocytes and neutrophils, as well as fibroblast growth factor 2 (FGF 2) and VEGF, hence facilitating the process of wound tissue vascularization. In cases of thrombocytopenia, macrophages and T lymphocytes present at the site of injury serve to compensate for the deficiency of platelet-derived growth factors (PDGFs) and commence the inflammatory phase (35, 38, 55–57).

### 2.2 Inflammatory Phase

Inflammation in wound healing involves a complex coordination of immune system cells and molecular interactions, including macrophages, neutrophils, lymphocytes, and signaling molecules. It begins after hemostasis, where blood arteries dilate due to coagulation and complement cascades, with Bradykinin and anaphylatoxins playing crucial roles (58). Anaphylatoxins increase blood vessel permeability, allowing monocytes and neutrophils to reach injury sites. They also stimulate mast cell production of histamine and leukotrienes, amplifying inflammation response. This enhances EC permeability, disrupting cell-to-cell connections and facilitating inflammatory cell movement at wound sites (59). Neutrophils, which are the primary leukocytes present in wounds, play a crucial role in combating infection by using several mechanisms, such as antimicrobial peptides, proteases, and reactive oxygen species (ROS). These entities possess a limited duration of existence and experience programmed cell death through mechanisms involving caspases and cathepsin D (59, 60). Neutrophils do not hinder wound healing, but their prolonged absence may lead to chronic nonhealing wounds. Monocytes, prevailing inflammatory cells at wound sites, migrate towards the injury site through the chemotactic process facilitated by other immune cells like neutrophils and keratinocytes. CCL2 chemokines direct monocyte migration, while other immune cells like neutrophils and keratinocytes also play a role (61). Monocytes migrate to wound sites and differentiate into macrophages, which is crucial for wound healing. Macrophages eliminate apoptotic neutrophils, present antigens, and produce cytokines and growth hormones. They also eliminate diseased cells and present antigens (62). Growth factors like TGF-α, TGF-β, bFGF, PDGF, and VEGF activate and recruit cells, promoting wound healing through proliferation, ECM formation, and angiogenesis. VEGF also promotes angiogenesis by eliciting the expression of LIGHT, a cytokine within the TNF-α family, in macrophages, potentially causing apoptosis (63). The coordinated interaction between immune system cells and signaling molecules is crucial for the systematic advancement of wound healing, including the initial inflammatory response as well as later stages of repair of tissues and remodeling. A comprehensive comprehension of these processes is of utmost importance in the development of efficacious treatment approaches for wound healing and the management of chronic wound diseases (54).

Macrophages play a crucial role in the process of wound healing since the lack or dysfunction of these cells dramatically impedes the closure of wounds. Research indicates that the depletion of macrophages during the process of skin wound healing might result in poor tissue disposal, a decrease in the number of fibroblasts, and a delay in the healing process. Nevertheless, it is important to note that inflammation is not an essential prerequisite for the process of wound healing of skin (30, 64). The findings of a study using PU.1 null mice have shown that these mice exhibited comparable wound repair rates to their wild-type littermates while notably lacking scar formation. These observations imply that the presence of inflammation may not be a need for the process of wound healing (61, 62). Studies show that modulating the inflammatory response can help reduce scar formation in wound healing. However, impaired macrophage activity at the wound site can hinder inflammation resolution, especially in diabetic wounds. This continuous inflammatory state can compromise wound closure and worsen scar formation, especially in diabetic wounds where the continuous inflammatory state prevents a resolving phenotype (54, 65, 66). Lipid mediators like protectins, resolvins, lipoxins, and maresins regulate inflammation and control wound healing. They elicit actions to resolve and counteract inflammation. B and T lymphocytes are crucial for wound healing as they initiate targeted immune responses against pathogens and foreign substances (62). Mast cells play a vital role in wound healing by triggering inflammation and resolving it through apoptosis, facilitated by interferon-c and TNF α. They also influence wound healing and fibrosis processes. However, their compromised healing in mice and their association with keloids and hypertrophic scars suggest the need for further research into their specific function and potential therapeutic applications (66). Understanding immune cell function in wound healing is crucial for developing effective therapeutic interventions, especially in chronic or impaired situations, to enhance closure and minimize scarring.

#### 2.2.1 Immune cells in wound healing

##### 2.2.1.1 Neutrophils

Neutrophils, comprising 50% to 70% of leukocytes in adults, are crucial in initiating inflammation at sites of acute inflammation or infection. They exist in a dormant state and have a lifetime of 8-12 hours while circulating in circulation and 1-2 days when residing in tissues. Macrophages play a crucial role in initiating the inflammatory phase at wound sites, triggered by stimuli such as growth factors, chemokines, and N-formyl peptides. The buildup of neutrophils at wound sites reaches its maximum during the early phase of inflammation and decreases after four days (67–70). Neutrophils in wounds facilitate the leukocyte recruitment cascade towards inflamed tissue, secreting chemoattractants, releasing ROS and antibacterial proteins, effectively eradicating infections (71, 72). Neutrophils regulate innate and adaptive immune responses through intercellular communication with chemokines, cytokines, and immune cells. Their presence of proteases enhances antibacterial efficacy and tissue penetration. However, the overproduction of proteolytic enzymes can damage receptors, growth factors, and ECM and impede vascular processes, highlighting the potential dangers of neutrophil overproduction (73).

Neutrophils, activated and producing neutrophil extracellular traps (NETs), are crucial in trapping and removing pathogens. They are discharged through suicidal and NETosis mechanisms, allowing neutrophils to survive and participate in tasks like phagocytosis. Prompt clearance of neutrophils is essential for inflammation resolution, as failure may lead to chronic wounds like venous leg ulcers, diabetic foot ulcers, and pressure ulcers (70, 72, 74, 75). This highlights the significance of prompt clearance for good wound healing and reduction of inflammation in situations both normal and pathological circumstances.

##### 2.2.1.2 Monocytes

Monocytes are essential for the human immune system, acting as a defense mechanism against infections (74). Originating from the bone marrow, they differentiate into macrophages and dendritic cells, each with distinct immune responses. Monocytes can be classified into three subsets: CD14++CD16− classical monocytes, CD14++CD16+ intermediate monocytes, and CD14+CD16++ nonclassical monocytes, each with unique functions, size, morphology, and transcriptional profiles (76).

Research using deuterium labeling has provided insights into the life cycle of monocytes in the bone marrow. Monocyte precursors differentiate into classical monocytes, followed by a postmitotic maturation phase (77). Their lifespan is around one day before apoptosis or circulation exit. A smaller percentage matures into intermediate monocytes, lasting four days (65). A significant proportion of intermediate monocytes transform into nonclassical monocytes, lasting seven days before cell death (78). Monocyte migration is a regular process, allowing them to persist in tissues, acquire antigen-presenting capabilities, and differentiate into macrophages (79). The dynamic nature and adaptability of immune responses are crucial in understanding monocyte-macrophage populations in skin tissue (65, 77–79).

##### 2.2.1.3 Macrophages

Following tissue injury, macrophages identify molecular patterns linked to damage or infections, activating monocytes and neutrophils to migrate toward the wound site. Monocytes secrete chemokines and cytokines, causing neutrophils to chemotaxis towards the wound location. Neutrophils expel their granules, attracting inflammatory monocytes that differentiate into macrophages (78). These immune cells have flexibility, allowing them to differentiate into diverse phenotypes based on the wound site’s conditions. The recognition of patterns initiates a series of interconnected reactions, including the synthesis of inflammatory chemicals and pro-inflammatory cytokines (80). Macrophages and neutrophils are essential components in wound healing, actively participating in the removal of pathogens, deceased cells, and tissue remnants. Both exhibit similar capabilities, including phagocytosis, killing intracellular mechanisms, and generating NETs (80, 81). After debris clearance, neutrophils collaborate with macrophages to terminate inflammation and restore homeostasis. The shift towards an anti-inflammatory state promotes tissue restoration and restructuring during wound healing (78, 82–84).

##### 2.2.1.4 Lymphocytes

###### 2.2.1.4.1 T Lymphocytes

Innate lymphoid cells (ILCs) are essential in the innate immune response, distinguishing them from T cells, B cells, and NK cells. The ILC family consists of three subsets: Group 1, NK cells responsible for secreting IFN-γ and TNF-α, and ILC2 cells involved in wound healing (85). The activation of ILC2s by IL-33 promotes reepithelialization and wound healing (86). Invariant NK T cells significantly impact skin wound healing by increasing IFN-γ production, releasing growth factors like VEGF and TGF-β, enhancing collagen accumulation, facilitating myofibroblast differentiation, and stimulating blood vessel formation. Invariant NK T cells also help mitigate neutrophil-organized inflammatory responses, expediting wound healing. These various innate lymphocyte subsets are crucial contributors to skin wound healing and the immune system environment (39, 87, 88).

###### 2.2.1.4.2 Cytotoxic T cells

Skin injuries trigger an immune response using pattern recognition receptors (PRRs), including TLR, NOD-like receptors, and C-type lectin receptors. This triggers the release of pro-inflammatory cytokines and the influx of macrophages and neutrophils (89). Skin wounds cause DCs to break down PAMPs and DAMPs, delivering them to naïve CD8+ T lymphocytes in draining lymph nodes (89). T cells differentiate into effector and central memory T cells, and apoptosis occurs when skin-homing receptor-expressing TEM moves to wound sites and releases immune mediators (90, 91).

Secondary lymphoid organs express lymph node-homing receptors by TCM cells, leading to their differentiation into TEM cells. These TEM cells travel to distal lymph nodes, establishing systemically immune memory. Local DCs move towards inflamed skin areas, offering antigens to skin-resident CD8+ Trm cells. This interaction leads to the proliferation of CD8+ Trm cells and the recruitment of effector memory T cells (TEM), contributing to pathogen clearance (92). CD8+ Trm is localized in the epidermis, serving as the first defense mechanism against reinfections (93, 94). The skin’s immune systems respond efficiently to external assaults, facilitating wound healing and establishing immunological memory. Understanding these physiological mechanisms could help develop therapeutic interventions for enhancing immune system responses and wound healing.

###### 2.2.1.4.3 Helper T cells

The skin’s immune system relies heavily on CD4+Foxp3+ Tregs, which regulate immune responses and contribute to the skin’s microenvironment (95, 96). These cells, which express CCR6 and CLA, move and aggregate in skin hair follicles, promoting wound reepithelialization and modulating inflammation. They also limit IFN-γ production and control inflammatory macrophages (39, 97).

Tregs, or regulatory T cells, persist in the skin after pathogen clearance and play a crucial role in maintaining long-term immunological control in healthy adult skin (98). CD4+ T cells secrete cytokines that induce inflammatory responses, maintaining immunological homeostasis and defense against infections. Their diverse roles highlight their importance in skin protection (99).

###### 2.2.1.4.4 B lymphocytes

B cells are essential in the humoral immune response, transforming into plasma cells that produce antibodies. They also transport antigens to T cells and regulate local immunological responses through the secretion of cytokines and growth factors (100). Studies have shown that mice with spleen deprivation experience a delay in wound healing after injuries (101). B cells treat wounds by generating antibodies against injured tissue and producing cytokines that repair wounds (102).

A study by Sirbulescu et al. found that mature B cells accelerate wound healing, reducing healing time by 2-3 days. This highlights the potential of B lymphocytes in wound healing. B cells are integral to the immune system and play a pivotal role in various immunological responses (103). Recent studies have highlighted the importance of antibodies in wound healing, as they generate antibodies, present antigens, and regulate immune responses by releasing cytokines. Understanding the multifaceted involvement of B cells in wound healing not only advances our understanding of immunity but also presents opportunities for treatments (6).

##### 2.2.1.5 Mast cells

**Figure 2:**
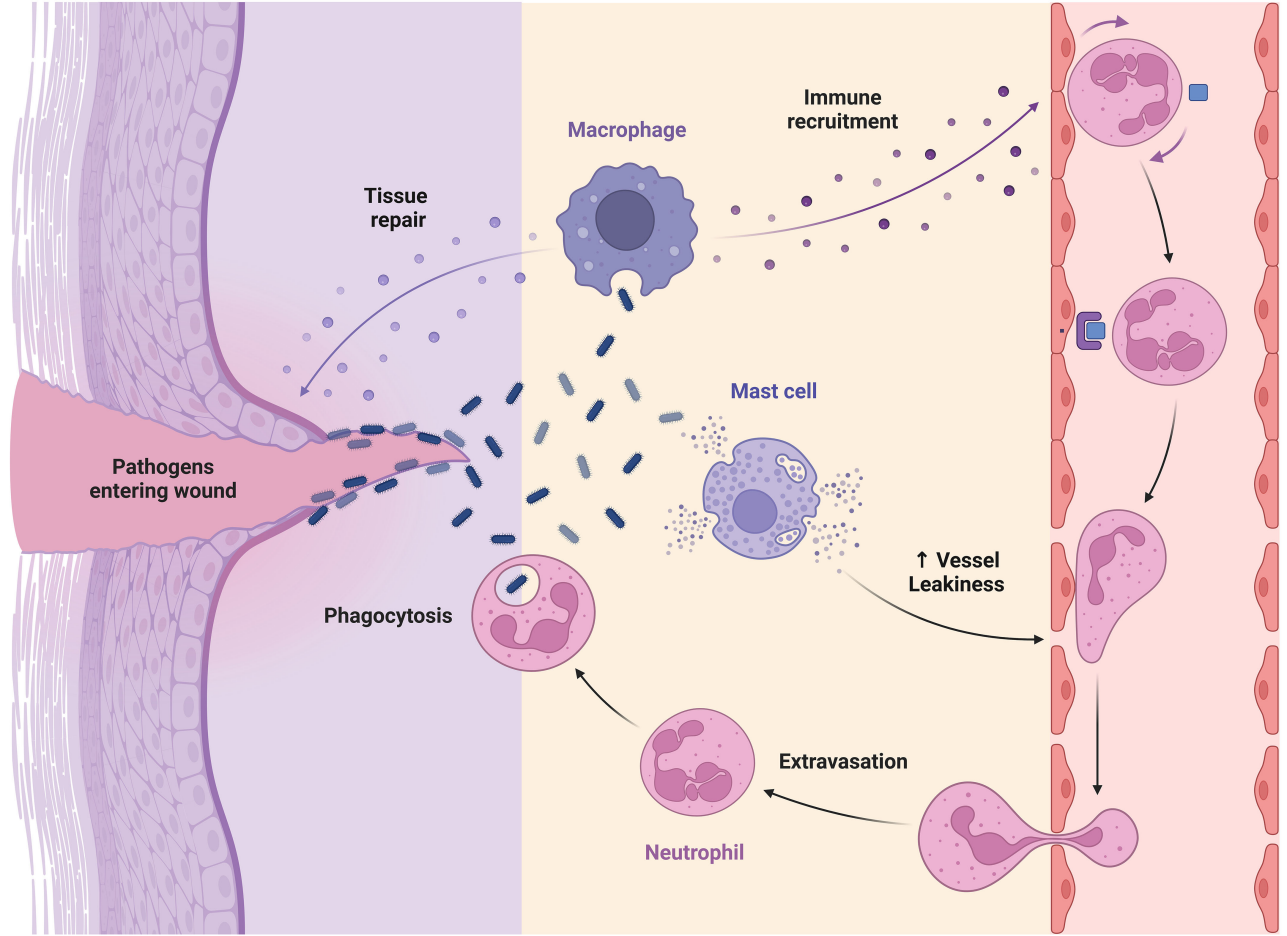
Role of macrophage, mast cell, and neutrophil in wound healing.

During wound healing, mast cells secrete antimicrobial peptides, enzymes, VEGF, and histamines, which promote neutrophil influx and vascular permeability, preventing skin infections (104). Histamine from mast cells encourages keratinocyte growth and re-epithelialization, while histamine and tryptase boost fibroblast proliferation and collagen production, contributing to wound contraction (6, 105). A model of fetal wound healing found that a rise in mast cell numbers is associated with skin fibrosis and scarring. In 15 embryonic day wounds, injection of mast cell lysate causes scar development, while deletion of mast cells lowers scar development in 18 embryonic day wounds (106). However, further characterization is needed to understand the precise processes involved in mast cell participation in scar development. Mast cells have functional variability depending on their tissue and microenvironment, leading to several subsets with unique activities in wound healing (**Figure 2**) (107). Further investigation is needed to understand the distinct functions performed by different subsets of mast cells in wound healing, particularly in cases with prolonged duration or compromised healing capacity.

##### 2.2.1.6 Dendritic cells

Skin-derived cells (pDCs) are essential in the immune system, facilitating T-cell responses and presenting antigens. Langerhans cells, first discovered in the 19th century, are derived from early myeloid progenitors and continue to exist into maturity (6, 108). They are responsible for exposing antigens to T cells inside the dermis and relocating to the discharging lymphatic system to initiate T-cell responses (109).

In the dermis of mice, there are two primary subtypes of resident DCs: CD11b DCs and CD103 DCs. CD103 DCs are capable of cross-presenting antigens, detecting deceased cells, and perceiving stimuli like F-actin and DAMPs, initiating immunological responses (110). The significance of skin-derived cells (pDCs) in wound healing has received less attention than their role in fighting infection. Researchers can use single-cell technology to understand better the roles of different types of immune cells in immune responses and wound healing. The classification of Langerhans cell types and dermal DCs is a subject of discussion (111).

#### 2.2.2 Apoptosis

Apoptosis, a regulated form of cell death, serves a critical function in the stages of tissue healing, as shown by its complex involvement in the resolution of these phases (112). The occurrence of apoptosis in inflammatory cells may be noticed as early as 12 hours after the occurrence of a lesion, as shown by the findings of Brown et al. in their research conducted in 1997. Additional investigations conducted on rats have demonstrated the occurrence of apoptosis in scab-inflammatory cells and myofibroblasts, which play a critical role in wound healing and tissue regeneration. This process was seen to begin around the twelfth day, reach its highest point on the twentieth day, and then resolve by the sixtieth day (113).

The research findings demonstrate a temporal association between the process of myofibroblast apoptosis and the healing of wounds, explicitly highlighting the role of stromal keratocytes (114). The initiation of cellular processes crucial for corneal wound healing is facilitated by apoptosis, whereas a delay in healing might occur due to delayed phagocytosis of apoptotic neutrophils by macrophages. The investigation also examines the involvement of apoptotic cells in the release of growth signals that induce cell proliferation. The fundamental determinant of wound healing is the process of apoptosis in immune cells, which prompts inquiries about the principal component (115).

##### 2.2.2.1 Apoptosis as the method of cellular elimination in wounds

The process of necrosis, which involves tissue healing and inflammation, is improbable to serve as the primary mechanism for lowering cellularity in wounds. The method of emigration is characterized by inefficiency and energy expenditure, hence making apoptosis the most rational strategy for reducing cell numbers throughout the stages of healing. Apoptosis serves as a ubiquitous mechanism for the elimination of unnecessary cells and tissues while concurrently mitigating the risk of inducing further inflammatory responses. The induction of apoptosis in inflammatory cells may be triggered by the presence of invading organisms and nonviable tissue. Similarly, fibroblasts engaged in the process of collagen deposition can also suffer apoptosis. Following the process of wound maturation, it has been shown that ECs and any residual fibroblasts undergo a gradual disappearance without any noticeable signs or symptoms (116).

Brown et al.’s mouse dermal wound investigation showed that apoptosis is essential for tissue healing. After the injury, inflammatory cells apoptosis within 12 hours, indicating epithelial factors inhibit inflammation. Clinicians have found that wound closure or grafting reduces inflammation (113).

Esmouliere et al. demonstrated that apoptosis reduces fibroblasts and aids wound healing. The shift from granulation to scar tissue, a crucial tissue healing step, was studied. Inflammatory cells of exposed wound scabs showed early apoptosis. The research found that myofibroblasts, important contractile cells in wound healing, undergo apoptosis beginning at day 12, peaking at day 20, and ending at day 60 (117). As the wound progressed, cells creating new blood vessels showed a comparable apoptotic pattern, indicating a coordinated reduction in cellularity. This indicates cell-to-cell communication. Covering a 10-day-old incision with a particular skin flap caused myofibroblast death within hours (118).

A comprehensive understanding of the mechanism by which apoptosis drives the decline in fibroblast and myofibroblast function is of utmost importance in the context of wound healing. Failure to regulate this process in a timely manner may result in persistent apoptotic dysfunction, aberrant collagen production, and the creation of excessive scar tissue (117–119). The use of growth factor therapy expedites the progression of apoptotic patterns and the healing of wounds, hence reducing the duration of the inflammatory phase. Animals with diabetes exhibit a delay in the healing process of wounds and an increase in apoptotic cells, which may be attributed to several processes associated with diabetes and variable degrees of damage in the healing process (118, 120).

#### 2.2.3 Autophagy

**Figure 3:**
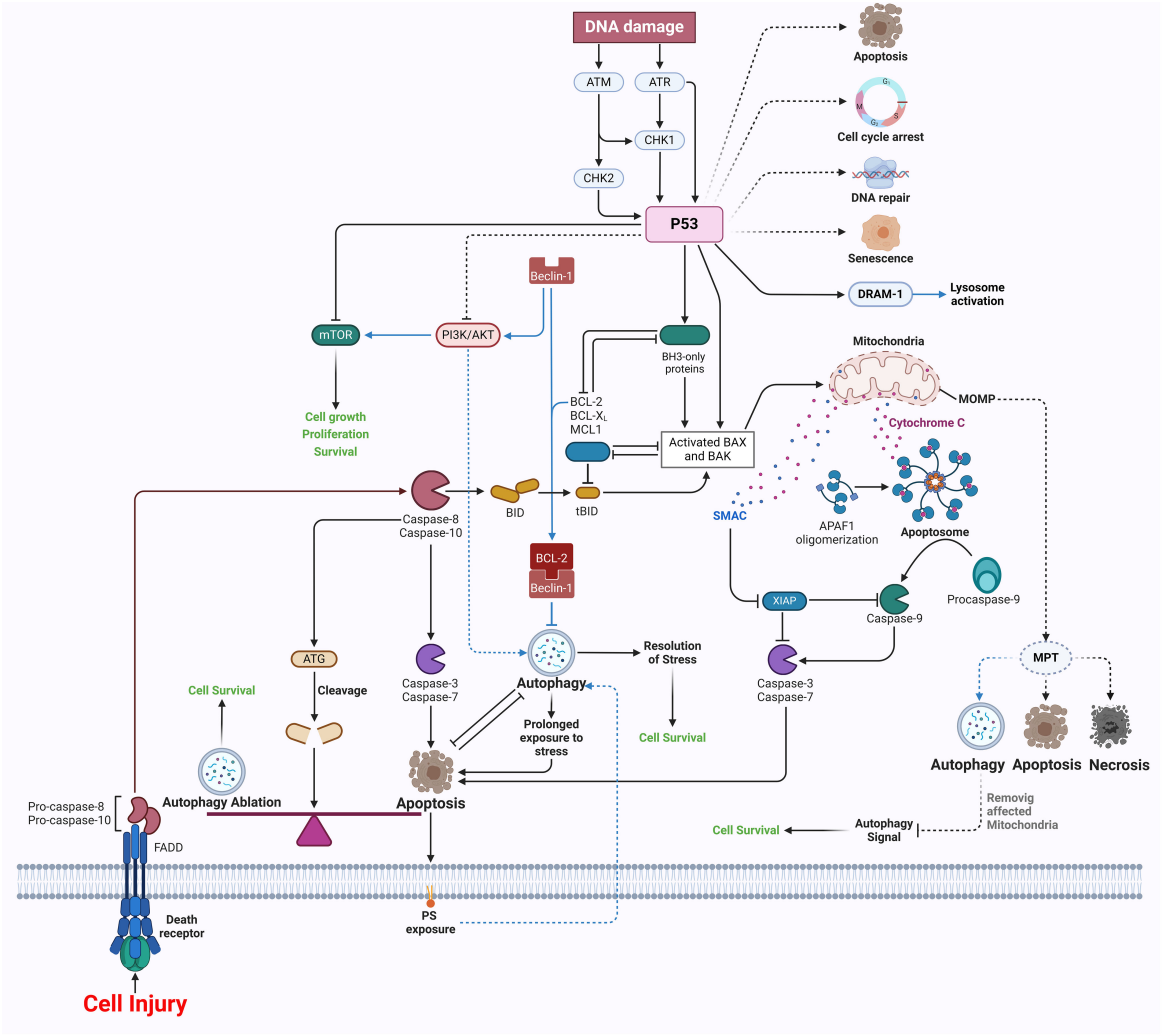
Apoptosis and autophagy in wound healing.

The cellular process of autophagy, which involves the degradation and recycling of cellular components, is known to have a significant impact on wound healing. It actively participates in several phases of the healing process, contributing to its overall efficiency and effectiveness (121). The early phase of inflammation after an injury serves to commence the process of wound healing by recruiting monocytes and neutrophils to the site of damage. Neutrophils are essential in the defense against microorganisms and the facilitation of inflammation by using antibacterial and proinflammatory processes such as phagocytosis, formation of ROS, degranulation, and the release of neutrophil extracellular traps (122). Autophagy is of great importance in the context of neutrophil-specific activities since it has been seen to enhance the phagocytic activity of human neutrophils when subjected to Streptococcus pneumoniae. The reduction of autophagy leads to a decrease in rates of phagocytosis. The investigation using mice that lack specific autophagy-related genes (Atg5/7) demonstrates a reduction in neutrophil degranulation and levels of ROS, therefore emphasizing the complex relationship between autophagy and neutrophil functionality (**Figure 3**) (123).

Moreover, the stimulation of autophagy enhances the creation of NETs. It increases the survival rate in mice afflicted with sepsis, therefore emphasizing its significance in the immune response. Furthermore, the impact of autophagy on *macrophages* is evident in the observation that the administration of 3-methyladenine amplifies their capacity to engulf pathogens, therefore emphasizing the intricate interplay between autophagy and macrophage functionality (121, 124). Moreover, the impairment of autophagy in macrophages results in their polarization towards the M1 phenotype, while the stimulation of autophagy favors the M2 phenotype, hence diminishing inflammation and facilitating tissue regeneration. However, the precise underlying mechanism remains ambiguous and needs additional research (125).

Recent research has demonstrated that MSCs have crucial functions in the process of tissue regeneration and wound healing. These functions include encouraging the growth of new blood vessels (angiogenesis), controlling inflammatory responses, and improving the formation of new epithelial tissue (126). Prior research has shown that controlling autophagy could be a successful approach to enhance the survival of MSCs and enhance the results of wound healing (127). A prior investigation discovered that palmitate stimulates the programmed cell death of MSCs by causing an increase in the levels of ROS inside the cells. However, the activation of autophagy, which occurs via the ROS–JNK–p38 MAPK signaling pathway, safeguards MSCs from undergoing apoptosis (128). Overexpression of hypoxia-inducible factor-1α enhances the survival of MSCs in low oxygen circumstances by stimulating autophagy via the suppression of PI3K/AKT/mTOR signaling (129).

Furthermore, the serine/threonine kinase aurora kinase A triggers autophagy by specifically targeting FOXO3a in order to safeguard adipose-derived stem cells from death caused by hyperglycemia. Other research has shown that blocking microRNA (miR)-34a enhances the effectiveness of MSCs in treating diabetic wounds by activating the sirtuin-1/FOXO3a pathway-mediated autophagy (130). Furthermore, An et al. demonstrated that autophagy inducer-pretreated MSCs injected subcutaneously stimulate VEGF production by triggering MSC-specific paracrine signaling via the ERK1/2 pathway, which in turn improves wound healing (121, 126).

#### 2.2.4 Ferroptosis

Ferroptosis is characterized by the absence of nucleosomal DNA condensate, chromatin condensate, and apoptotic body development, distinguishing it from conventional necrosis features such as organelle enlargement and cytoplasmic rupture (131). Ferroptosis is a metabolic anomaly characterized by a diminution in the size of mitochondria, a reduction in cristae density, and an increase in membrane density. Notably, the nucleus remains unaffected in terms of its size. The condition is based on a metabolic imbalance that relies on iron, resulting in the excessive buildup of lipid ROS and subsequent cell death. Cysteine metabolism is the fundamental process (132). The cellular uptake of cystine occurs through a cysteine-glutamate antiporter known as system xc-, while the efflux of glutamate takes place. Thioredoxin reductase 1 (TrxR1) facilitates the reduction of cystine, leading to its conversion into glutathione (GSH) via the enzymatic actions of GCL and GSS (133). The lipid-repairing enzyme GPX4 is responsible for the conversion of phospholipid hydroperoxides into non-peroxide forms, therefore effectively suppressing their pro-oxidative properties. The interference of Erastin with the uptake of cystine by the system xc- transporter results in the depletion of glutathione, thus inhibiting the activity of GPX4. Consequently, this disruption leads to the buildup of ROS in lipids, an excess of iron, and an enhanced vulnerability to ferroptosis (134). The condition of iron overload leads to the occurrence of cellular ferroptosis, a process in which external Fe^3+^ molecules bind to ferritin, subsequently entering the cell via the transferrin receptor 1 (TFR1) and undergoing reduction to Fe^2+^ facilitated by STEAP3. The introduction of an excessive amount of Fe^2+^ ions into the Fenton reaction leads to the occurrence of ferroptosis (133, 135).

There is a strong correlation between ferroptosis and skin wounds (136). Multiple investigations have shown that the use of ferroptosis inhibitors may enhance the healing of diabetic wounds by suppressing the process of ferroptosis. For instance, when ferrostatin-1 (a substance that inhibits ferroptosis) is applied directly to a wound in rats with diabetes, it may speed up the healing process by blocking ferroptosis via the activation of the PI3K/Akt signaling pathway (137). Furthermore, several studies have shown that the use of DFO, an alternative form of ferroptosis inhibitors, might enhance the healing process of diabetic wounds. Gao and colleagues discovered that the simultaneous use of DFO and hydroxysafflor yellow A in a hydrogel may expedite the recovery of diabetic wounds in rats by promoting angiogenesis (138).

**Figure 4:**
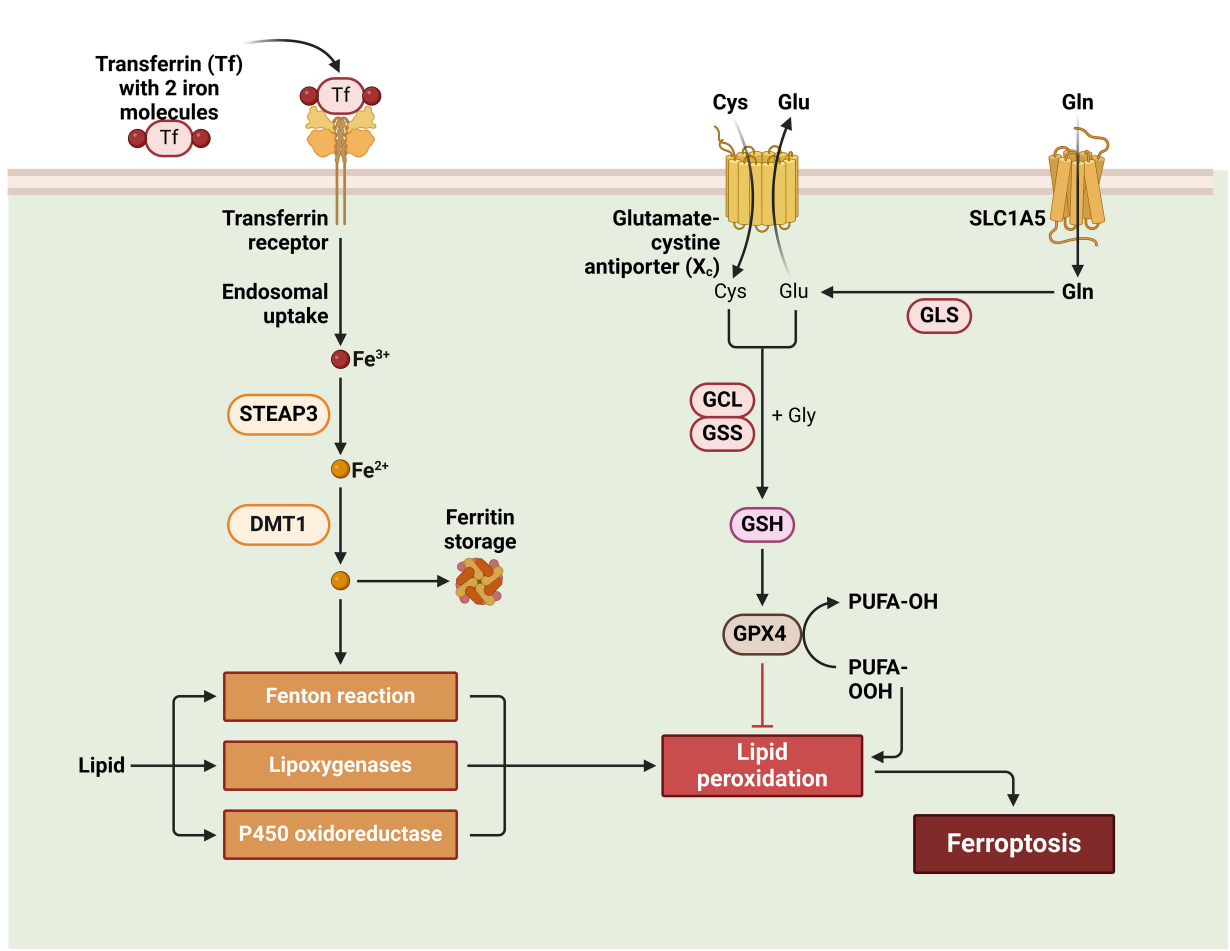
Ferroptosis in wound healing.

Excessive exposure to radiation may injure the nearby blood vessels and lead to the development of angiosclerosis. This occurs due to the detrimental effects on the structure and functions of proteins and DNA, resulting in a delay in the healing process of wounds (139). Gan et al. have shown that injecting plasma-derived exosomes (RP-Exos) locally may enhance the healing of rat irradiation wounds by increasing the growth, movement, cell division, and survival of fibroblasts. Additionally, it has been shown that RP-Exos can interfere with the ferroptosis pathway, hence preventing ferroptosis in irradiated fibroblasts (140). In addition to irradiation treatment options for tumors, excess exposure to UV radiation, particularly UVA and UVB, may also result in UV-induced cutaneous wounds. Kavita Vats and her colleagues have shown that an overabundance of UVB radiation may trigger inflammation and the death of human keratinocytes by generating ferroptosis. This process can be suppressed by ferrostatin-1. In addition, an overabundance of UV radiation may lead to a deficiency in GSH, which in turn disrupts the balance of redox reactions in the body (**Figure 4**) (136, 141).

**Figure 5:**
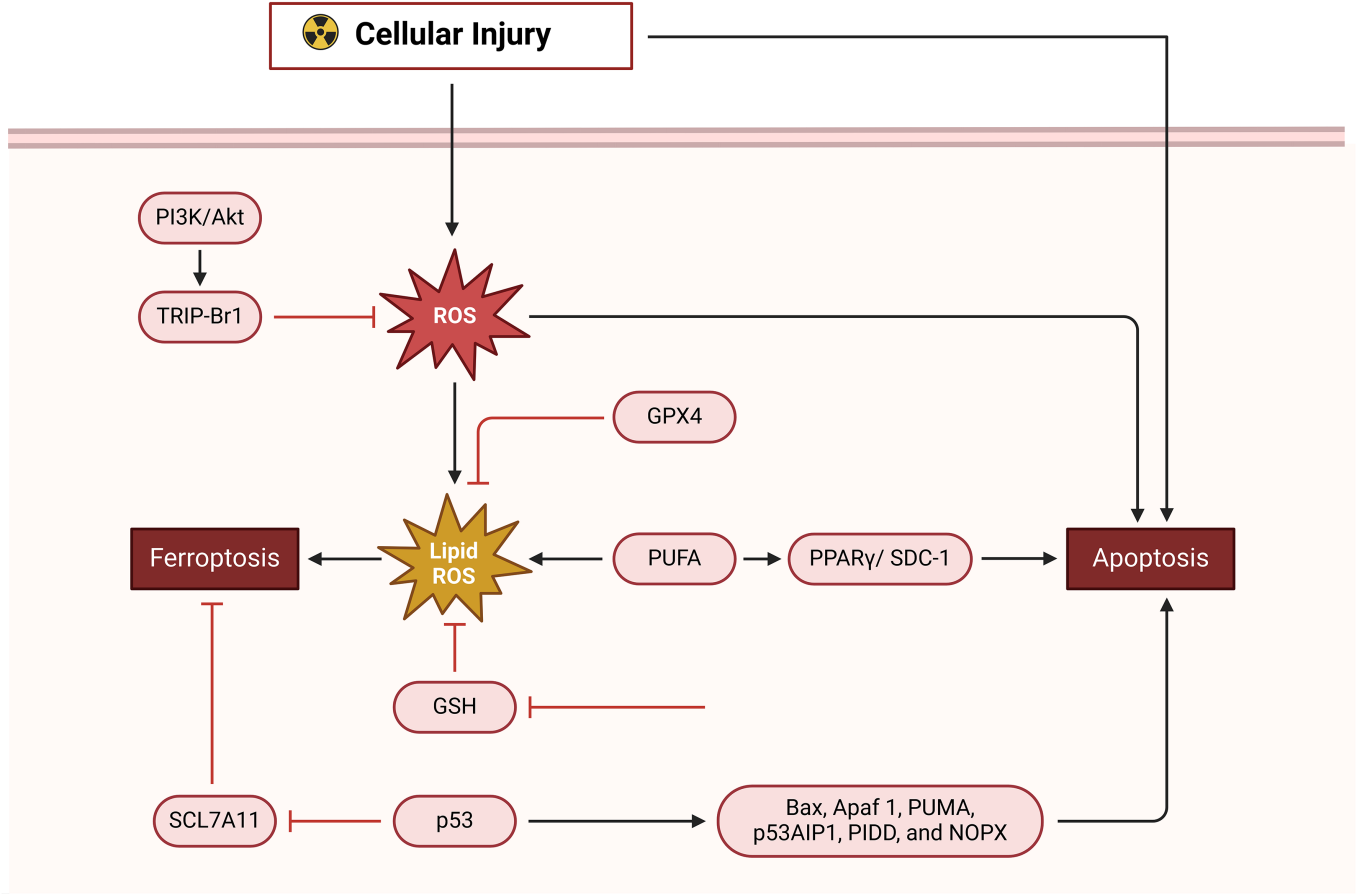
Apoptosis and ferroptosis in wound healing.

Ferroptosis, which involves lipid metabolism, requires lipid peroxidation. Free polyunsaturated fatty acids (PUFAs) are converted into PUFAs-phosphatidylethanolamine (PUFAs-PE), especially arachidonic acid (AA) and adrenaline, which are substrates for lipid peroxidation. ACSL4 and LPCAT3 are critical enzymes in this pathway (136, 142, 143). ACSL4 and LPCAT3 regulate Ferroptosis. LPCAT3 creates PUFAs-PE from PUFAs-CoA, while ACSL4 creates PUFAs-CoA. Ferroptosis results from PUFAs-PE oxidation and lipid peroxidation. ACSL4 is expressed more in ferroptosis-sensitive cells like HepG2 and HL60. MLE cells without LPCAT3 are more resistant to ferroptosis, lowering cell death (**Figure 5**) (136, 144).

#### 2.2.5 Pyroptosis

Chronic wounds are characterized by an overactive inflammatory response, which hinders the normal progression of the wound-healing process. This disruption ultimately results in the development of scars since it interferes with the sequential progression of the four phases of wound healing (145). The NLRP3 inflammasome, which is primarily located in cutaneous epithelial tissues, plays a pivotal role in orchestrating the immunological response of the organism. Recent studies have demonstrated that the administration of mulberry leaf and fruit extract (MLFE) has the potential to augment the process of wound healing, with a specific focus on its impact on the NLRP3 inflammasome. The combination of MLFE with mulberry leaf extract has been shown to exhibit enhanced anti-inflammatory and anti-obesity properties (146). The research proposes that MLFE can restore NLRP3 inflammasome levels in individuals with obesity, which might facilitate prompt wound healing. This finding underscores the possible associations between MLFE and obesity (147, 148).

TFNAs, DNA nanomaterials, promote corneal and skin healing with their angiogenic, antioxidant, anti-inflammatory, anti-fibrotic, and low-toxicity functions (146, 149). TFNAs heal diabetic wounds faster, decrease skin fibrosis, and block pyroptotic pathways (150). Bioactive glass (BG) may work for wound healing and soft tissue restoration. It controls the Cx43/ROS signaling pathway to suppress EC pyroptosis and improve wound healing (151). BG inhibits caspase-1 activation and perforation, slows ROS generation, and regulates connexin 43 expression, promoting blood vessel development and wound healing (148, 152).

The interaction between inflammasome, pyroptosis, and wound healing is complicated and needs additional study (153). Novel carriers and Chinese herbal extracts are being tested for therapeutic use. Recent studies have shown that the TFNAs, NLRP3 inflammasome, and BG improve healing and reduce problems. Understanding these relationships may help design chronic wound treatments and individualized wound healing techniques (148, 154).

**Figure 6:**
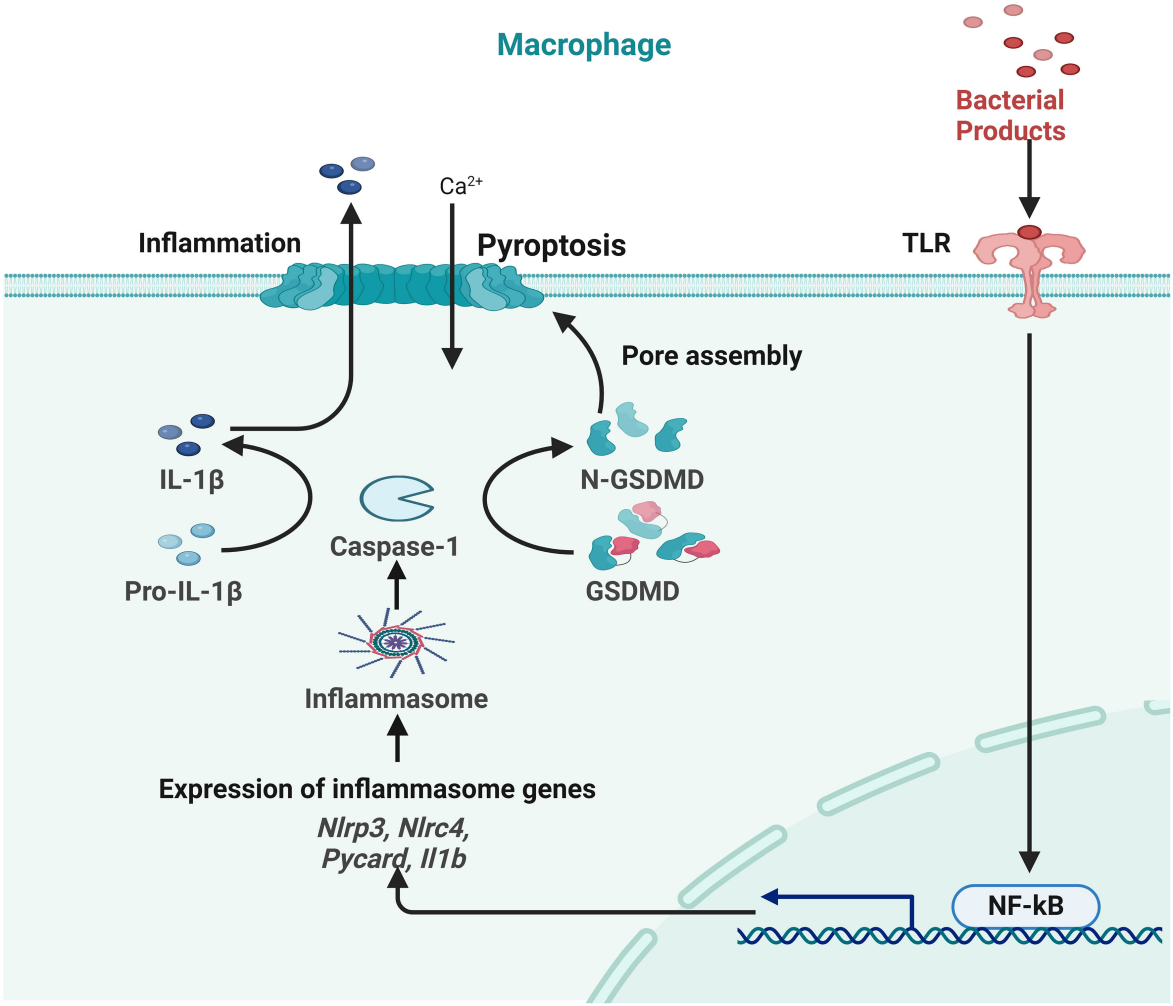
Role of pyroptosis in inflammation during skin wound healing.

Pyroptosis can be induced with caspase-1, NLPR3, ILβ and IL18. The natural healing process of wounds depends upon NALP3 signaling. Elevated pro-inflammatory cytokines, including TNF-α, IL-1β, and IL-6, facilitate wound healing. Multiple research studies show that the signaling pathway of the NALP3 inflammasome is of significant importance in the process of skin wound healing (155). Research conducted both *in vitro* and *in vivo* has shown that TFNAs improve corneal transparency, hasten the process of wound reepithelialization, and have a beneficial effect on the healing of corneal epithelial wounds (150). TFNAs have advantages not only in promoting the healing of corneal wounds but also in facilitating the healing of skin wounds. The research findings demonstrated that the use of TFNAs expedited the skin wound healing process and mitigated scarring. The first study indicates that nanophase materials with nucleic acid biological characteristics have the potential to expedite wound healing and minimize scarring. These findings suggest that TFNA might be used to facilitate the regeneration of skin tissue (156). Studies have shown that TFNA suppresses the pyroptotic pathway, which in turn lowers levels of inflammatory cytokines and increases the amount of collagen in the skin. Following treatment with TFNA, levels of NLRP3 inflammasome and pro-caspase-1 were found to be decreased. This suggests that the inflammasome was reduced together with the active form of caspase-1, which led to a subsequent decrease in N-terminal GSDMD levels. According to the findings, TFNA has both anti-inflammatory and anti-fibrotic properties, although it does not cause cytotoxicity (152). Pro-caspase-1, caspase-1, NLRP3, GSDMD, and a number of other proteins associated with the pyroptosis and inflammasome signaling pathways were shown to be present in this research. In summary, TFNAs have essential scientific relevance for skin wound healing, and it has been established that they are strongly associated with pyroptosis and inflammasome pathways. This suggests that TFNAs may play a role in the activation of these pathways (**Figure 6**) (148, 152).

#### 2.2.6 Cuproptosis

Tsvetkov et al. discovered “cuproptosis,” a cellular death process characterized by the transportation of Cu to mitochondria (157). The finding mentioned above has inspired investigations into the control of mitochondrial copper and its possible implications in cancer treatment. Previous studies have shown a connection between the toxicity of elesclomol in cancer cells and many parameters, such as the levels of ferredoxin-1 and the rates of mitochondrial respiration, which are influenced by the availability of Cu (157).

Cuproptosis, a particular type of cell death that is reliant on Cu and occurs inside mitochondria, exhibits distinct characteristics when compared to well-established processes such as ferroptosis, apoptosis, or necroptosis. FDX1, an essential participant in this mechanism, functions as a ligand for elesclomol, facilitating the conversion of Cu^3+^ ions to Cu^2+^ ions and subsequent release inside the matrix of mitochondria (158). Research indicates that FDX1, metabolic enzymes, and targets of lipoylation proteins within the PDH complex mediate Cu ionophore toxicity. Posttranslational lipoylation adds lipoic acid to mitochondrial proteins, reducing Cu-induced cell death. FDX1-dependent cancer cells require lipoic acid pathway components for survival, and modifications reduce metabolic enzyme lipoylation, making some cells susceptible to elesclomol-induced cuproptosis (157, 159, 160).

Cuproptosis refers to the phenomenon in which mitochondrial Cu undergoes a process of substitution with proteins, hence playing a crucial role in elucidating the physiological significance of Cu in the human body. Cu is often located inside the matrix of mitochondria and is integrated into the assembling process of cytochrome c oxidase. Copper shortages have been shown to be associated with the occurrence of developmental problems. However, it has been observed that the administration of elesclomol can effectively mitigate these defects (161). The use of Cu metalloallostery, a mechanism that governs several cellular processes, such as proliferation and autophagy, has been under scrutiny due to its possible harmful effects. Cuproptosis is a phenomenon characterized by cellular demise, which is intricately associated with copper biology (162, 163).

According to Tsvetkov’s study findings, cuproptosis might potentially play a role in the development of Wilson’s illness, which is defined by an abnormal buildup of Cu in the body. Increased Cu concentrations in liver cells trigger the activation of Cu metalloallostery, which can remove impaired mitochondria and alleviate cuproptosis (157). The buildup of metals generated by ions affects cellular homeostasis, hence impacting the stability of proteins. Histone H3 can attach to Cu and function as a Cu reductase, therefore indicating the presence of many protein binding sites that may accommodate exchangeable Cu ions (164). Cuproptosis has the potential to induce cellular demise via the process of copper-mediated aggregation (162, 165).

Cuproptosis, controlled cell death using copper buildup and reactive oxygen species, is associated with cancer neurological, and cardiovascular problems. Recent research suggests it may aid wound healing (162, 165). Research shows that wound healing may be accelerated by upregulating cuproptosis-related genes and delayed by suppressing it. Cuproptosis is an essential process in the context of wound healing as it facilitates the elimination of impaired cells, recruits inflammatory cells, and governs the orchestration of new tissue generation. Nevertheless, the specific contributions of the subject in question continue to be a topic of continuous scholarly investigation, whereby the possible advantages and disadvantages are contingent upon the particular circumstances (165).

#### 2.2.7 Necrosis

Necrosis takes place when an illness remains untreated or when harm to tissue reaches an irreversible state. The condition can advance into the deeper layers of tissue, impacting the integrity of bone tissue and possibly resulting in the development of bacteremia and sepsis (7). Skin necrosis may be due to either extrinsic causes or vascular blockage. Necrosis is a pathological condition that is distinguished by cellular or tissue demise, often leading to a discoloration of the skin in shades of purple, blue, or black. Frequently seen necrotizing disorders include necrotizing fasciitis, ecthyma, and meningococcemia (166).

### 2.3 The proliferative phase

#### 2.3.1 Neovascularization: granulation tissue

The proliferative stage of wound healing is a complicated process that includes the creation of neovascularization, re-epithelialization, granulation tissue, and immune system modulation. This phase of wound healing occurs after the inflammatory stage of wound repair. Granulation tissue is predominantly activated fibroblasts, as described by Alexis Carrel and John Hunter. These fibroblasts produce new ECM, which helps to contract wounds and forms cellular and structural elements (30, 167).

Neovascularization is necessary for the healing of wounds, the transport of nutrients, the preservation of oxygen balance, the multiplication of cells, and the regeneration of tissue (30). During the early stages of embryonic development, endothelial progenitor cells (EPCs), also known as angioblasts, give rise to the first blood vessels. Angiogenesis is the primary process that leads to the development of new blood vessels during adult tissue healing. This process involves the activation of local microvascular ECs in response to hypoxia and growth factors such as PDGF and VEGF. Proliferation, migration, and the development of capillaries are all processes that ECs go through (168).

ECs and pericytes are involved in the angiogenesis procedure. These cells react to hypoxic injury settings by beginning the cycle of angiogenesis, which includes tearing down the ECM and generating blood vessels (169). Pericytes maintain the integrity of these vessels. Granulation tissue may also be formed owing to the participation of circulating progenitor cells and several subtypes of fibroblasts in the formation process. Understanding these systems is necessary for the development of therapeutic strategies for wound healing (6).

#### 2.3.2 Neovascularization: endothelial cells

Angiogenesis refers to the physiological process through which new blood vessels are formed, mainly via the involvement of ECs (170). The initiation of the procedure is influenced by a multitude of variables, such as the presence of proteolytic enzymes and growth factors (171). Enzymes such as these facilitate the migration of ECs via clots that are rich in fibrin and fibronectin, hence promoting the repair of injured regions. ECs also exhibit a response to hypoxia and engage in interactions with perivascular cells, therefore emphasizing the complicated structure of their stimulation. This mechanism has a critical role in both physiological and pathological contexts (172). VEGF, FGF, PDGF-B, TGF, and angiopoietins stimulate EC to migrate and proliferate to start angiogenesis (171–173). Some ECs become lead tip cells and extend filopodia toward pro-angiogenic growth factors, while others become trailing stem cells (174).

The Notch signaling pathway is of significant importance in the determination of ECs as either tip or stalk cells. The regulation of this pathway is governed by VEGF, which is synthesized by several cellular populations inside the wound microenvironment. VEGF-A, which belongs to the VEGF family, plays a crucial role in the process of angiogenesis by directing tip cells towards regions of higher concentration gradients and promoting the proliferation of stalk cells in a concentration-dependent manner. The complicated interaction among ECs, growth factors, and signaling pathways exemplifies the sophisticated and orchestrated nature of vascular development (171, 175, 176).

#### 2.3.3 Neovascularization: circulating progenitor cells

EPCs have garnered significant scientific attention since their first characterization in 1997. Initial investigations have shown that HSCs, as well as EPCs, are involved in the process of blood vessel regeneration. EPCs undergo a sequential three-step mechanism in order to get into ischemic tissues (177). This process involves their mobilization from the bone marrow, subsequent navigation via the circulatory system, and eventual integration into sprouting endothelium (178). Nevertheless, new research has cast uncertainty on the actual function of EPCs in the processes of ischemia-responsive vasculogenesis and blood vessel restoration (179). In mouse models, it has been shown that circulating progenitor cells do not undergo differentiation into ECs at sites of damage or tumor formation (180).

The identification and characterization of circulating progenitor in blood vessels have posed significant challenges, mostly stemming from the complexities associated with their proper isolation from the circulatory system or the surrounding microenvironment (178, 179, 181). The use of single-cell technologies has excellent potential in elucidating the identification and function of cells involved in the process of wound healing, hence facilitating a more accurate comprehension of the mechanisms behind blood vessel regeneration and repair. The acquisition of this information has the potential to enhance the efficacy of wound healing mechanisms and perhaps facilitate the development of specific therapeutic approaches for vascular restoration (6, 181).

#### 2.3.4 Fibroblast

Fibroblasts are an assortment of cells located in the dermal layers, demonstrating notable versatility and serving a range of functions (6, 182). Fibroblasts respond to soluble extracellular signals, including growth factors and cytokines, by undergoing activation, leading to cellular proliferation and the modulation of metalloproteinases. During the process of healing a wound, fibroblasts that have reached maturity move toward the granulation tissue, therefore initiating the production of collagen and substituting the temporary fibrin matrix (183, 184). Myofibroblasts are generated, leading to an increase in the accumulation of collagen and the initiation of contraction of the wound. Fibroblasts possess the ability to perceive both the magnitude and direction of mechanical loads, after which they convert this sensory input into appropriate adaptive responses (183, 185). To illustrate fibroblast behavior, consider the intermediary filament vimentin, which inhibits fibroblast growth, promotes the accumulation of collagen, and stimulates keratinocyte mesenchymal reepithelialization and differentiation. It is essential for the study of wound healing procedures and, possibly, for the development of focused treatments in wound care to have a firm grasp on the complex mechanisms governing fibroblast activity (186).

#### 2.3.5 Myofibroblasts

**Figure 7:**
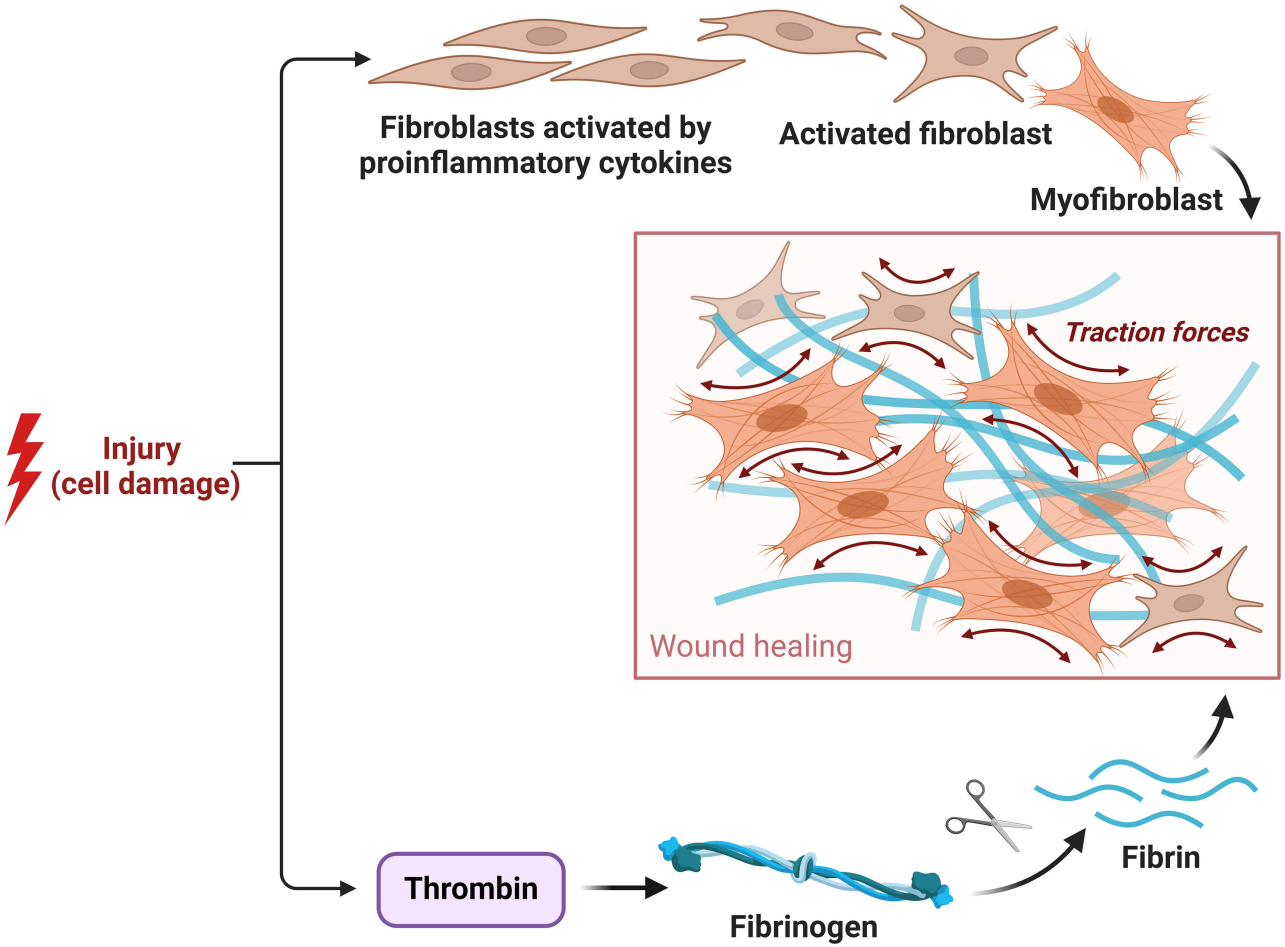
Role of fibroblast and myofibroblast in the proliferative phase.

The process of wound healing encompasses wound contraction. This phenomenon leads to a reduction in the surface area of the wound and an improvement in the mechanical strength of the surrounding tissue. The procedure mentioned above converts migratory fibroblasts into myofibroblasts that express α-SMA, leading to the deposition of ECM and facilitating the process of wound healing (187). Myofibroblasts, which exhibit contractile properties similar to smooth muscle cells, contribute to wound healing by depositing ECM components such as collagen type I and III. They achieve wound contraction by connecting to polymerized fibronectin and collagen fibrils (188). The ability to exhibit alternative protein contractility and induce apoptosis upon the restoration of the integrity of tissues has been observed (6, 189). Nevertheless, under pathological circumstances such as hypertrophic scarring, the presence of myofibroblasts might endure, hence playing a role in the excessive formation of scar tissue. The strategic targeting of myofibroblasts has significant potential in the advancement of therapeutic approaches for fibrosis and damage, rendering them a critical focal point for therapeutic interventions. Comprehending the dynamics and control of myofibroblasts is essential in the progression of wound healing procedures and the formulation of tailored medicines to enhance results in healing wounds and fibrosis management (**Figure 7**) (6, 189).

#### 2.3.6 Re-epithelialization

The epidermis, an integral layer of the skin, serves as a vital protective barrier from external factors and plays a critical role in the process of wound healing. The structure in question is comprised of many distinct layers, including the basal, granular, spinous, and stratum corneum layers. Its main constituents are keratinocytes, which are interconnected by desmosomes. The basal layer is in direct association with the basement membrane, housing many components such as hair follicles, immune cells, sebaceous, and sweat glands. The presence of stem cells inside the epidermis plays a vital role in the processes of repair and maintenance (6, 43, 190). Lineage tracing investigations have been conducted to discern the presence of stem cell populations inside different skin structures, such as the interfollicular epidermis (IFE), hair follicles, sebaceous glands, sweat glands, and melanocytes. The concept known as the EPU explains the processes of regeneration and repair. In this model, slow-cycling stem cells located in the basal layer of the epidermis generate transit-amplifying cells via a process of asymmetric proliferation (191). iPSCs exhibit a phenomenon known as inflammatory memory, which facilitates their ability to mount rapid reactions when encountering subsequent challenges. The turnover of epidermal appendages, such as sebaceous glands, hair follicles, and sweat glands, is a continuous process that involves lineage-restricted stem cells dedicated to the maintenance of their respective cell types. Gaining a comprehensive understanding of the dynamics and characteristics of these cells provides valuable insights into the processes behind wound healing. Such insights have the potential to pave the way for the development of precise wound-healing techniques and regenerative medicines (6, 43, 192–194).

#### 2.3.7 Peripheral nerve repair

When a person has traumatic damage to a peripheral nerve, the homeostatic function of their skin is disrupted. Collateral reinnervation and nerve regeneration are both necessary steps in the procedure of repairing neurological functionality (195). The peripheral nervous system in adults is capable of regenerating nerve activity via the process of growing back the terminals of myelinated nerve and rejoining the wounded nerve (196). Schwann cells, monocytes, macrophages, and fibroblasts are essential players in the process of regenerating nerves. Schwann cells are distinguished by their exceptional plasticity, which enables them to redifferentiate into progenitor-like cells after damage. This facilitates the rebuilding of axons (39, 195, 197).

The process of Schwann cell differentiation initiates the secretion of monocyte chemoattractant protein-1, PAP-III, IL-1α, and IL-1β, which in turn pull monocytes/macrophages to the location of damage. Macrophages are known to secrete VEGF and HIF, hence facilitating the process of angiogenesis (198). The newly reorganized vasculature functions as a supportive structure for Schwann cell cords, reducing the guidance of axon development. The complex nature of this process guarantees the restoration of nerve function after an injury, and comprehending these processes is of utmost importance in the development of specific therapeutic approaches for traumatic damage to the peripheral nerve (199).

### 2.4 The remodeling phase

The procedure of wound healing encompasses a prolonged phase of tissue remodeling or maturation, which may last for many months or possibly extend over years. The period, as mentioned above, has a substantial impact on the probability of scar formation and wound recurrence. The remodeling phase encompasses the regression of neovasculature and the remodeling of tissue from granulation into scar tissue (52). The presence of collagen III is first seen in granulation tissue, and over time, it undergoes a progressive replacement by collagen I. During the progression of wound remodeling, the myofibroblasts present in the granulation tissue actively produce matrix metalloproteinases (MMPs) and their inhibitors (TIMPs), which play a crucial role in selectively degrading particular ECM components (52, 200). An aberrant production of TIMPs and MMPs may give rise to an asymmetry in ECM modification, which may, therefore, contribute to the development of chronic wounds (52, 117). Macrophages are of significant importance in the process of wound remodeling as they are responsible for the degradation of excessive ECM and the phagocytosis of debris (201).

During the remodeling stage of wound repair, the blood vessels that are generated during the process of angiogenesis exhibit increased permeability and a deficiency in tight intercellular junctions. This characteristic facilitates the infiltration of immune cells into the wound site. The process of neovessel pruning is essential for the development of stable and well-perfused blood vessels, which involves the programmed cell death of ECs. Vessel pruning may also be influenced by the process of re-epithelialization (30). ECs possess negative feedback systems that regulate their response to VEGF (202). These processes include the stimulation of intracellular Sprouty proteins and Vasohibin. During the advanced phase of wound healing, endothelial cells demonstrate the expression of CXCR3, which subsequently hinders the process of endothelial tube formation. The comprehension and manipulation of these mechanisms have the potential to result in specific therapies, enhanced results in wound healing, and decreased scarring (202).

#### 2.4.1 Difference between healed and physiological tissue: scarring

In response to injuries, the human body undergoes a series of physiological processes that include wound healing and the subsequent creation of scars. Scars are tissue characterized by an effective neo-formation process, which involves the replacement of lost tissue with an ECM that is mainly composed of fibronectin and collagen types I and III. Specific components of the skin, including hair follicles, subepidermal appendages, and glands, have little or no regenerative capacity after significant damage (203). The scar tissue matrix, which is denoted by granulation tissue, is distinguished by a considerable concentration of fibroblasts, capillaries, macrophages, granulocytes, and collagen fibers (204). During the first stage of scar formation, known as the primordial scar tissue phase, the process of angiogenesis is still ongoing but not fully developed. The predominant cell type during this phase is fibroblasts (205). The process of scar development concludes during the remodeling phase, commencing on day 21 and continuing for one year subsequent to the occurrence of an injury. During this particular stage, the ECM constituents experience continuous modifications, characterized by the replacement of collagen III with type I collagen and the involvement of myofibroblasts in the process of wound contraction. Excessive use of scarring results in a disruption of the equilibrium between the processes of biosynthesis and degradation, which subsequently gives rise to a prolonged inflammatory phase, an extended proliferative phase, and a diminished remodeling process (204–206). Abundant microvessels are present in hypertrophic scars, and aberrant scarring is also characterized by alterations in the ECM and epithelial tissue (7).

#### 2.4.2 Role of stem cells in wound healing

The predominant kind of stem cells used in medical applications are adult stem cells, which are obtained from several sources, such as bone marrow and adipose tissue (207). According to previous research, MSCs exhibit anti-inflammatory and immunomodulatory characteristics during the inflammation phase while also promoting the activation of fibroblasts, keratinocytes, and ECs during the proliferative phase. This cellular response ultimately leads to an expedited healing of wounds (207). Research has shown that the use of MSC treatment has the potential to decrease the duration of wound healing, minimize wound contraction, enhance the formation of new blood vessels (angiogenesis), and expedite the process of epithelialization (208, 209). The administration of stem cell treatment may occur by topical or systemic means. Topically, MSCs can be applied in the kind of spray, aiding in the retention of these cells throughout the wound (210). In order to enhance the retention of stem cells inside a wound, it has become common practice to provide these cells with suitable support or scaffold material, such as collagen or skin substitutes. This phenomenon contributes to the preservation of cellular functionality and promotes the movement of cells inside the wound site (35, 211).

## 3 Pharmacological management

### 3.1 Conventional approaches employed for wound healing

Traditional methods of wound care include a range of interventions, including surgical interventions, non-surgical therapies, and the use of pharmaceutical agents to facilitate the healing process of skin wounds. Surgical interventions include debridement, skin grafts, and skin flaps, while non-surgical modalities involve the use of topical medications, wound dressings, and skin replacements (212). Surgical debridement is often regarded as the preferred method for wound treatment because of its high efficacy and expeditiousness. However, it is essential to acknowledge that this approach is not without its inherent hazards, including the administration of general anesthesia and potential tissue damage (213).

Skin grafts are often used in surgical procedures to control wounds, taking into consideration the specific characteristics and causes of the wound. The two main categories of skin grafts are split-thickness skin grafts and full-thickness skin grafts. Split-thickness grafts are appropriate for treating tiny lesions that only include damage to the epidermis. Full-thickness grafts are a valuable treatment option for extensive and deep wounds due to their ability to facilitate healing in both the epidermal and dermal layers, leading to the formation of healed tissue without visible scarring (7). Nevertheless, the use of allografts is restricted due to the scarcity of available donor skin and the need for a wound bed with adequate vascularization. Skin grafts may be categorized as autografts, allografts, and xenografts, depending on the source of the donor. Autografts are often regarded as the preferred method for skin restoration since they include the use of healthy skin obtained from the same individual to restore the integrity of the skin effectively. Allografts have the advantage of readily accessible donor skin and the capacity to be stored for extended periods for potential future use. In contrast, xenografts refer to skin grafts acquired from distinct species and then implanted into human recipients (214). Nevertheless, both allografts and xenografts are subject to some restrictions, including immunological rejection, the potential for disease transmission, the occurrence of painful healing, and the formation of scars (7, 215).

Traditional non-surgical methods for treating wounds include a range of interventions, such as the use of dressings for wounds, topical medications, scaffolds/hydrogels-based skin transplants, and skin replacements. The objective of these treatments is to achieve equilibrium in moisture levels, address issues related to inflammation and infection, and regulate the processes of contraction and re-epithelialization (216). The skin is most often targeted for medication delivery via topical formulations, which include gels, emulsions, pastes, creams, foams, lotions, and sprays. Topical antibiotics, such as silver sulfadiazine and neomycin, have shown efficacy in combating bacterial infections. Nevertheless, it is advisable to halt their use in order to prevent the development of hypersensitivity and allergic responses (217). Topical products have been subjected to experimentation in order to assess the efficacy of growth factors in promoting wound healing. Growth factors have significant potential in treating skin wound healing. They stimulate cell proliferation, promoting the formation of new tissue and wound closure. They also enhance angiogenesis, blood supply, and nutrient and oxygen delivery to the healing tissue (218). Growth factors can modulate inflammation, reduce inflammation, and promote tissue repair (219). They regulate the synthesis and remodeling of the extracellular matrix, enhancing tissue strength and integrity (220). They facilitate the migration and differentiation of cells, promoting the differentiation of specialized cell types for tissue repair (221). Growth factors may also reduce scar formation and improve the aesthetic outcome of wound healing (222). Some growth factors have antimicrobial properties, reducing the risk of wound infection and promoting a more favorable healing environment (223). Advances in delivery systems, like hydrogels, nanoparticles, and scaffolds, allow for controlled delivery of growth factors at the wound site, optimizing their therapeutic effects while minimizing adverse effects (224). Overall, growth factors hold promise as a therapeutic approach to enhance skin wound healing by promoting faster and more efficient tissue repair processes (225).

However, the restricted bioavailability of growth factors, caused by their quick elimination from the wound site, imposes limitations on their practical use. Nanoparticle-encapsulated growth factors are now being used in topical applications as novel methods to enhance collagen production and angiogenesis (226).

Cytokeratins, specifically KRT16 and KRT17, play a crucial role in skin wound healing. They are upregulated during the process of wound healing, providing structural support to keratinocytes, the predominant cell type in the epidermis. They also regulate cell proliferation and differentiation, with KRT16 and KRT17 promoting keratinocyte proliferation, essential for the formation of new epithelial tissue (227). Cytokeratins may also modulate the inflammatory response during wound healing, interacting with immune cells and signaling molecules involved in inflammation. Changes in cytokeratin expression patterns have been proposed as biomarkers for monitoring wound healing progression and assessing therapeutic interventions’ effectiveness (228). Dysregulation of cytokeratin expression or function has been linked to the pathogenesis of chronic wounds, where impaired re-epithelialization contributes to delayed healing. Understanding the roles of cytokeratins in wound healing may offer insights into novel therapeutic strategies for chronic wound management (229).

Natural biomaterials, particularly keratins, play pivotal roles in wound healing, offering promising therapeutic avenues in both healthy individuals and those with conditions such as diabetes. Keratin-based biomaterials possess inherent biocompatibility and bioactivity, serving as effective scaffolds for cell attachment, proliferation, and differentiation, thereby promoting tissue regeneration. They also exhibit modulatory effects on the inflammatory response, fostering a shift towards a pro-healing environment, which is particularly relevant in chronic wounds observed in diabetic patients (229). Keratin-based dressings help maintain moisture balance in the wound environment, preventing excessive drying or exudate accumulation and promoting cell migration, proliferation, and ECM deposition (230). They also promote angiogenesis, a hallmark of chronic wounds, by providing cues for endothelial cell proliferation and migration, enhancing blood vessel formation and tissue perfusion in the wound bed (218). Keratin-based biomaterials have been investigated for their potential to reduce hypertrophic scars or keloids, which can impair function and aesthetics. They can promote more organized ECM deposition and reduce excessive fibrosis during the healing process. By being biodegradable and biocompatible, keratin-based biomaterials are suitable for various wound healing applications, including in diabetic patients with compromised healing capacity (**Table 2**) (238).

**Table 2:** Summary of the differences in wound healing in healthy and diabetic subjects.

Aspect of Wound Healing	Healthy Subjects	Diabetic Subjects	Ref.
Inflammatory Response	Well-coordinated, resolves efficiently	Prolonged and dysregulated, chronic inflammation impedes healing	(231)
Angiogenesis	Robust and timely angiogenic response	Impaired angiogenesis, reduced blood vessel formation	(232)
Cell Proliferation	Enhanced proliferation of keratinocytes and fibroblasts	Reduced proliferation, delayed re-epithelialization	(233)
Extracellular Matrix (ECM)	Proper ECM deposition and remodeling	Abnormal ECM composition, increased fibrosis, and collagen deposition	(234)
Growth Factor Signaling	Effective growth factor signaling cascades	Impaired growth factor signaling, decreased responsiveness	(235)
Wound Contraction	Efficient wound contraction and closure	Impaired wound contraction, delayed closure	(236)
Infection Risk	Lower risk of infection due to intact skin barrier	Higher risk of infection due to compromised immune function	(237)
Scar Formation	Minimal scarring, efficient resolution	Increased risk of hypertrophic scarring or keloids	(237)

The acronym TIME, which stands for Tissue, Infection, Moisture, and Epithelial, serves as a complete framework for wound care, including several elements that impede the healing process (215, 239). The framework has four distinct components, including tissue assessment, infection evaluation, moisture management, and epithelium edge development. The primary objective of TIME is to analyze devitalized or non-self-material in the wound bed, evaluate the origin and treatment of infection, manage wound exudate, and check the advancement of edges as well as surrounding skin status (240). The comprehension of the relationship between TIME and the molecular biology of injuries has resulted in significant progress in the field of chronic wound care interventions and technology (7).

### 3.2 Wound physical therapy

#### 3.2.1 Negative pressure wound therapy

Negative pressure wound treatment (NPWT) is a wound healing technique that leverages the application of differential vacuum or suction to augment the healing process. The use of this treatment modality is often seen in the management of both acute and chronic wounds, including pressure ulcers, diabetic foot ulcers (DFUs), postoperative incisions, lower extremity wounds, traumatic wounds, infected wounds, burns, necrotizing fasciitis, infected sternal injuries, and post-skin grafting interventions. Significant advancements have been seen in individuals suffering from vascular lower leg ulcers and DFUs who possess adequate blood circulation (241, 242). NPWT devices are comprised of three essential components, as described in the literature (243). These components include a porous substance that fills the wound site, a drainage port that is coupled to a vacuum pump under regulated conditions, and an adhesive film dressing that effectively seals the wound. The porous substance often used is polyurethane foam, while the adhesive dressing is composed of polyurethane owing to its occlusive qualities (241, 244, 245).

NPWT facilitates the process of wound healing by using four main mechanisms: microdeformation, macrodeformation, elimination of wound fluid and decrease of edema, and modification of the wound microenvironment (242). Notwithstanding its efficacy, the use of this intervention has inherent dangers and possible consequences. There are three primary problems associated with this condition, including infection, bleeding, and the retention of foam dressing (241, 246). Bleeding occurs as a result of physical trauma to the underlying tissues, a condition that may be further exacerbated by factors such as necrotic tissue, infection, or coagulopathy. The use of NPWT in near proximity to accessible blood vessels, nerves, organs, or anastomotic sites is discouraged due to the heightened likelihood of fistulae development (245, 246). It is recommended to provide treatment for infections prior to the administration of NPWT. Additionally, it is essential to note that the non-dissolving nature of the dressing materials used in NPWT may potentially lead to complications such as bleeding or infection caused by foam retention. Additional concerns include the presence of discomfort during dressing changes and the occurrence of patient allergies to the adhesive bandage or foam substance (241, 243, 245, 247).

Enterocutaneous fistulas, which were formerly regarded as contraindications for non-operative percutaneous wound closure, are currently being managed with favorable outcomes. The device achieved fistula closure in a majority of the documented patients, namely those with low-output fistulas, comprising almost two-thirds of the total sample size of forty (248). Trauma patients, as well as those with abdominal compartment syndrome, are managed by using an open abdomen technique with the use of a negative pressure wound therapy device. This approach serves as a temporary measure by providing coverage, eliminating intraabdominal contaminants and exudates, and reducing visceral edema (249).

The use of NPWD closure has yielded substantial advancements in the management of severe orthopedic injuries. In the past, significant injuries to the extremities were managed by the use of extensive debridement, which necessitated the use of free flaps for rapid wound covering. The NPWD generates a hermetically sealed and safeguarded environment that effectively eliminates swelling and accumulation of blood outside blood vessels, hence enhancing blood flow and optimizing the preservation of the area around the wound. Serial debridements are conducted, and ultimate rebuilding is carried out in a stable wound in a planned manner (250). The contraindication of exposed tendons, joints, or bones has been reconsidered due to the formation of granulation tissue over these structures, which enables the possibility of skin grafting if deemed essential (251).

#### 3.2.2 Oxygen therapy

Historically, the two most critical oxygen-based treatments for wound care were known as hyperbaric oxygen therapy and topical hyperbaric oxygen therapy. However, recent developments in CDOT and Transdermal Oxygen Therapy have led to the introduction of a novel category of oxygen-based wound healing devices. These devices provide persistent treatment of wounds with oxygenation and are part of the oxygen-based wound care industry. Although these techniques promote wound healing in a manner that is analogous to one another, there are significant technical and medical distinctions between them (252).

##### 3.2.2.1 Systemic hyperbaric oxygen therapy

HBOT is a medical intervention in which a patient inhales pure oxygen at a pressure more significant than that of the surrounding atmosphere. This treatment has been shown to enhance the process of neovascularization, promote the production of ECM, and reduce inflammation (252). The use of this treatment has been shown to be advantageous in the management of late radiation tissue damage, acute wounds, chronic ulcers, and burns (241, 253). Neovascularization is initiated by an upregulation of ROS and RNS at the local level, leading to the activation of growth factors such as TGF-β, VEGF, and angiopoietin 2 (252). HBOT has been shown to induce the activation and specialization of circulating SPCs originating from the bone marrow, leading to the formation of blood vessels through the process of vasculogenesis. HBOT has been shown to enhance the production of fibroblast growth factors, which subsequently promotes the migration and proliferation of fibroblasts. The augmentation of oxygen levels serves as a stimulus for the proliferation of fibroblasts, leading to an increased production of collagen and subsequent enhancement of tissue tensile strength. In conclusion, HBOT has been shown to effectively decrease inflammation by reducing edema, inhibiting pro-inflammatory cytokines, promoting macrophage chemotaxis, enhancing leukocyte bactericide activity, and blocking neutrophil β2 integrin (251, 253, 254).

##### 3.2.2.2 Topical hyperbaric oxygen therapy

The progression of HBOT into THOT involves the use of a chamber that is placed around a wounded region to avoid leaks and to be filled with oxygen at high flow rates to provide a rich source of oxygen at the surface of the wound. When compared with HBOT, THOT is associated with a reduced risk of adverse effects, may treat a wider variety of therapeutic patients, and can be used in a wider variety of locations at a cheaper cost. However, in comparison to HBOT, it does not have the identical oxygen potential since collagen formation and cross-linking are lower, and the pace at which wounds are closed is slower. The effects of THOT are comparable to those of HBOT, and it was shown to speed healing in wounds that are persistent (255).

##### 3.2.2.3 Continuous diffusion of oxygen therapy

CDOT devices offer a convenient and portable solution for supplying a continuous stream of oxygen to wound sites. This technology serves as an alternative to THOT and HBOT methods. The delivery of oxygen at atmospheric pressure and slower flow rates to the wound bed necessitates the use of a wet dressing to facilitate diffusion. The term “THOT,” although characterized by high flow rates, is associated with intermittent treatment and necessitates a humid atmosphere (256). Although research is scarce in this area, several studies have demonstrated that THOT may have potential benefits in the healing of DFUs, ulcers associated with sickle cell disease, as well as in the treatment of stubborn and painful wounds when used as an adjuvant (255, 257).

## 4 Innovative strategies for wound healing

Healing wounds is a complicated process at the moment because various kinds of wounds have distinct molecular and cellular processes that must be addressed. Our knowledge of wound assessment and treatment methods has been significantly expanded as a result of developments in technology, which has resulted in a transition away from the use of traditional dry dressings and toward the use of modern moist dressings, growth factor therapies, responsive dressings, tissue-engineered skin, nanotherapeutics, gene therapy, and stem cell therapy. The road to individualized wound healing has been paved with the introduction of cutting-edge technologies such as 3D bioprinting, platelet-rich plasma treatment, and ECM-based techniques. However, the treatment of chronic wounds is still a problem, which calls for the advancement of unique and imaginative treatment methods that take into account their efficacy, the benefits-risks equilibrium, and the affordability and efficacy of the options (258).

### 4.1 Nanotherapeutics-based strategies

The process of wound healing may be complicated for doctors to manage. Still, nanotechnology has swiftly generated nanomaterials that are employed in medical, pharmacy, chemical manufacturing, and even in the military. Because of their one-of-a-kind physicochemical features, these materials produce effects that are localized to their surfaces and have a limited scale. The rising need for nanomaterial coverings such as nanofibers, hydrogels, and films has sped up the advancement of these materials, which has led to their increased use in a wide variety of disciplines (259). Advancements in nanotechnology and biomedicine have facilitated the use of nanomaterials in the healing of wounds. These materials have shown their efficacy in several aspects of the wound healing process, including hemostasis, antibacterial activity, inflammatory modulation, and promotion of cell proliferation. The augmentation of these characteristics may be accomplished by using certain technologies, such as the integration of nanoparticles with sodium alginate and gelatin, to produce a composite hydrogel (259). The antibacterial effectiveness may be enhanced by incorporating antibiotics into nanomaterials (260). The use of bionic nanofiber scaffolds has been shown to effectively mimic the ECM of the skin, hence facilitating the process of adhesion of cells and proliferation (261).

Nanoparticles are of significant importance in the context of wound healing applications, as they include inherent characteristics that expedite the process of wound healing. Additionally, researchers have been actively involved in the development of bioengineered drug delivery systems, which aim to provide sustained and regulated release of therapeutic molecules for enhanced wound healing outcomes. Metal and metal oxide nanoparticles, polymer nanomaterials, and several other nanotherapeutics have been extensively applied to the treatment of chronic wounds owing to their inherent benefits. Metallic nanoparticles, including gold, silver, zinc, and zinc oxide, have antibacterial characteristics that promote the process of wound healing (262). In addition to the substances mentioned above, cerium, nitric oxide, bioactive glass, and carbon-based nanoparticles are also known to possess inherent bioactivity.

Nevertheless, it is essential to consider the potential toxicity associated with metal-based nanotherapeutics prior to their *in vivo* use. The reduction of hazards in metal nanotherapeutics may be achieved by optimizing the size and characteristics (263). Polymeric nanostructures are now being used in the field of healing wounds and regeneration of the skin. The inclusion of antibiotics and growth factors inside polymeric nanoparticles has the potential to mitigate wound infection and expedite the process of wound healing (264). **Table 3** provides a summary of a complete list of nanotherapeutic-based techniques for the management of acute and chronic wounds. These strategies make use of inorganic as well as organic nanomaterials.

**Table 3:** Nanotherapeutics used for the management of wound healing.

Type of Nanomaterials	Adjuvants/Drug Association/Therapeutic Agents/Growth Factors	Model	Findings	Reference
**AuNPs**	Keratinocyte growth factor	HDFs: human epidermal keratinocytes (HEKs)	AuNPs were shown to enhance keratinocyte growth and accelerate wound healing.	(260)
**AuNPs**	Gallic acid, protocatechuic acid, and isoflavone	Rats	AuNPs enhanced dermis and epidermis thickness by suppressing MMP-1 and promoting angiopoietin-2, VEGF, and collagen.	(265)
**PLGA-liposome nanofibers**	MicroRNA 145 (miR-145) and platelet-derived growth factor (PDGF)	Rats	Improved wound healing via increased vascularization and more minor wounds.	(266)
**AgNPs**	Curcuma	*Streptococcus* *pyogenes*	Microorganism growth decreased; fibroblast cell proliferation and migration increased.	(267)
**AgNP hydrogels**	Gelatin and sodium alginate	Rats	Significantly decreased wound size.	(268)
**Poly (lactic-co-glycolic acid)-polyethylenimine nanoparticles**	Nitric oxide (NO)	Rats	Strong bactericidal effect against MRSA bacteria accelerated wound healing.	(269)
**Gelatin nanofibers**	anionic drug and hydrotalcite	NIH 3T3 fibroblast cell line	Faster wound healing and significant antibacterial activity	(270)
**Poly (ethylene terephthalate)** **(PET) nanofibers**	Anionic antibiotics piperacillin/tazobactam	*P. aeruginosa*-infected mouse	Low bacterial load, high efficiency, and sustained delivery	(271)
**Poly (l-lactic acid) (PLLA) nanofibers**	Dimethyloxalylglycine and silica nanoparticles	Rats	Accelerated collagen deposition and increased neo-vascularization and re-epithelialization	(272)
**Chitosan hydrogels**	Silver nanoparticles	*E. coli* and *S. aureus*	Promotion of antibacterial activity improved healing	(273)
**Nanofibrous** **membrane**	Chitosan-polyvinyl alcohol	Mice fibroblast cells; rat traumatic model and mice diabetic model	Upregulated growth factors, including TGF-β and VEGF	(274)
**Poly (lactic-co-glycolic acid)/gelatin** **(PLGA)/gelatin nanofibers**	Liraglutide (Lira)	Rats	Faster wound healing, better collagen synthesis and alignment, and more dense blood vessel growth	(275)
**Polyvinyl alcohol nanogels**	Cerium oxide nanoparticles	Rats	Antimicrobial activity and rapid healing	(263)
**TiO2 nanotubes**	IL-4	RAW 264.7 murine macrophage cells	Slowly releasing IL-4 during the first stage allowed M1 activation	(276)
**Poly-(1,4-phenyleneacetone** **dimethylene thioketal)**	SDF-1α	Mice	Induction of wound vascularization, improved wound healing	(277)
**ZnO NPs**	Alginate/acacia	Rabbits	Excellent biocompatibility, enhanced collagen and calcium deposition, fibroblasts, and reduced inflammatory cells, promoting faster wound healing.	(278)
**Photoluminescent gold nanodots**	Antimicrobial peptide and 1-dodecanethiol	Rat	Improved antimicrobial action and collagen deposition	(279)
**SiNPs**	Curcumin	human dermal fibroblast cells	Antimicrobial and antibiofilm actions improved using curcumin SiNPs as a photosensitizer.	(280)
**Chitosan nanoparticles**	Rebamipide	Rats	Improved re-epithelialization and faster wound healing	(281)
**Nanobioglass**	Chitosan hydrogel	Rats	Added to human whole blood *in vitro* shortened blood clotting time and formed stable blood clots *in vivo*.	(280)
**Liposome with silk fibroin hydrogels**	Essential fibroblast growth factor (bFGF)	Mice	Promoted wound closure and vascular vessel regeneration	(282)
**Collagen mats**	Inorganic polyphosphate (polyP)	Rat and Human	Reduced wound area, faster re-epithelialization, and healing	(283)
**GO scaffold**	Polyhydroxybutyrate-co-hydroxyvalerate copolymer, Fe_3_O_4_ NPs	Mouse fibroblast cells *in vitro*	Effective against gram- (–)ve bacteria strains, promoting wound constriction.	(284)
**Elastic liposomes with hyaluronic acid**	EGF, PDGF-A, and IGF-I	Rats	Reduced wound size, better-quality skin permeation	(285)
**Chitosan/Collagen blended nanofibers**	Curcumin	Rats	Reduced wound size, improved healing	(286)
**Polycaprolactone** **(PCL) nanofibers**	Alfalfa	Human dermal fibroblast	Increased epidermal keratinocyte and dermal fibroblast proliferation *in vitro*	(168)
**Solid lipid nanoparticles**	Serpin A1 (A1) and host defense peptide LL37	Rats	Increased wound closure and enhanced anti-inflammatory efficacy.	(287)
**Peptide dendrimers**		Rats	Smaller wound size, improved wound healing	(288)
**Silk fibroin nanoparticles**	Resveratrol	Mice	Lower intracellular ROS, type M2 macrophage polarization, restored colonic epithelial barriers, and decreased inflammation.	(289)
**Fibrin nanoparticles**	Keratinocyte growth factor	Rat	Smaller wound size, improved wound healing	(290)

The use of various nanomaterials in the regulation of the inflammatory state shows promise in the treatment of chronic wounds, making nanotherapeutics a potentially effective method. Burn wounds exhibit an upregulation of inflammatory mediators such as TNF-α, IL-6, and IL-1β. Conversely, diabetic wounds have a considerable expression of IL-18. Nevertheless, previous studies have shown that the application of silver nanoparticles and polymeric nanofibers resulted in a decrease in inflammatory markers, but it did not exhibit significant effects on IL-18 levels. This suggests that these interventions may have limited efficacy in reducing inflammation in the context of diabetic wounds (260, 262, 291). The identification of the inflammatory phase presents a challenge because of its overlap with the proliferative stages. Hence, there is an urgent requirement to produce nanomaterials that possess remarkable anti-inflammatory characteristics in order to facilitate the immunoregulation of many forms of chronic wounds. A plausible approach might include the use of immunomodulatory nanomaterials to identify distinctive indicators that demarcate the shift from the inflammatory phase to the proliferative phase. Nanotherapeutic-based techniques provide a potential avenue for the clinical management of chronic wounds. These approaches demonstrate notable antibacterial properties, effectively mitigate bacterial medication resistance, diminish the inflammatory phase, and expedite the wound healing process.

Nevertheless, a detailed summary of their processes has yet to be undertaken. This study critically examines the probable mechanisms and recent advancements in the use of nanoparticles for wound healing. It specifically focuses on the potential toxicity associated with these materials and identifies the present limits in clinical use and fundamental research (258).

### 4.2 Stem cell therapy-based strategies

Cell therapy encompasses a range of methodologies that use viable cells for therapeutic objectives, with the intention of rectifying, substituting, or reinstating the physiological functionality of a compromised tissue or organ. Stem cells are a kind of cells that have not yet undergone differentiation and can self-renew and develop into precursor or progenitor cells of one or several distinct cell lineages (292). The application of stem cells from adults is prevalent in the field of regenerative medicine. This is primarily because of their accessibility via *in vitro* growing techniques and the absence of ethical dilemmas (293). Bone marrow is well recognized as a prevalent source of adult stem cells that play a crucial role in the regeneration or repair of several tissues, such as the cartilage, bone, tendons, heart, and skin (294). Cell-based treatment for prolonged wound repair involves the use of many processes, including the interaction and impact of growth factors, the management of inflammatory procedures, and the activation of immunological processes to expedite re-epithelialization and vascularization (295). In the past few years, there have been many clinical and preclinical investigations that have shown significant effects on the improvement of wound healing quality by the use of stem cells (296). The beneficial effect of stem cell-based wound healing is primarily ascribed to its capacity to release pro-regenerative cytokines as well as growth factors, which facilitate skin regeneration in the management of chronic wounds (294, 297). Autologous stem cells possess remarkable differentiation capabilities, promote the formation of new blood vessels, and are generally well-tolerated by patients, exhibiting minimal adverse responses. The primary therapeutic objective of stem cell-based treatment is to specifically address the enhancement of wound healing quality in the context of wound care (298).

The primary sources of stem cells used for wound healing and skin regeneration are adult MSCs, ESCs, and iPSCs, which have been more recently investigated. The use of ESCs in wound healing has been limited owing to ethical problems that are linked to their use (299). MSCs play a crucial role in all four stages of wound healing, including facilitating the migration of cells to the site of injury, supporting the formation of new blood vessels (angiogenesis), releasing growth factors and cytokines, and aiding in the process of re-epithelialization. In the first human trial, researchers used MSCs generated from bone marrow to treat severe skin burn injuries, which were afterward followed by skin grafting. The study documented notable enhancements in neo-vascularization and pain alleviation (300). According to a research investigation conducted on burn injuries, the application of BMSCs into the wound site resulted in a reduction in wound contraction, modulation of EC activity, and an enhancement of angiogenesis (301). In a previous investigation, a comparable research endeavor used a biodegradable collagen membrane known as Coladerm, together with autologous bone marrow-derived MSCs and skin fibroblasts, to address the issue of chronic non-healing wounds, namely diabetic ulcers. The findings of this study revealed a notable reduction in wound size and an improvement in vascularization after a combined treatment period of 29 days (302).

According to recent research, it has been shown that exosomes formed from stem cells, which include growth factors and cytokines, can expedite the process of wound healing by several mechanisms such as facilitating cell proliferation, migration, re-epithelialization, angiogenesis, and activating signaling pathways (303). Adipose-derived MSCs are often used in the context of wound healing owing to their ease of access, limited invasiveness, and absence of ethical constraints (304). Umbilical cord mesenchymal stem cells (UC-MSC) have shown encouraging therapeutic outcomes in the treatment of chronic wounds in individuals with diabetes, exhibiting increased cellular proliferation and collagen deposition (305, 306). The use of MSCs in conjunction with natural substances has shown augmented therapeutic attributes in third-degree burn injuries, exhibiting heightened levels of angiogenesis, wound closure, and re-epithelialization as compared to control groups. The transplantation of human Wharton’s jelly stem cells (HWJSC) with acellular dermal matrix has been shown to result in improved healing of burn wounds, according to previous studies (307, 308).

Conversely, Cell reprogramming technology encompasses the process of transforming mature somatic cells into iPSCs. This technique has considerable promise for many applications within the pharmaceutical sector, clinical settings, and laboratory environments. iPSCs have comparable attributes to ESCs, but they are not exempt from ethical considerations. Autologous cells have the potential for expansion and use, hence circumventing problems associated with immune rejection (302). The use of iPSCs in producing human autologous cells for progressive persistent wound therapy and degenerative skin illnesses has been extensively explored via *in vitro* and *in vivo* research conducted on mice models, revealing significant promise (309). iPSCs may be produced using many methodologies, one of which involves the transduction of somatic cells with a mixture of reprogramming factors. Nevertheless, the process of reprogramming adult somatic cells and promoting differentiation in the target cell line has proven to be challenging (310, 311). The utilization of iPSC methodologies can generate a substantial quantity of human autologous cells, rendering them suitable for implementation in genome editing methodologies. Autologous iPSC has the potential to serve as a viable and enduring therapeutic option for addressing chronic injuries that arise from genetic predisposition (303). Nevertheless, there is a need for more comprehension of these cellular classifications to ensure the safeguarding of patient well-being (298).

## 5 Challenges and future prospectives

In recent years, there has been a significant focus on the enhancement of cutaneous wound healing. This has led researchers to investigate the fundamental processes involved and devise novel treatment strategies to improve the overall quality of wound repair. The use of nanotherapeutic-based methodologies has garnered significant interest in the medical community due to their potential efficacy in the treatment of many forms of chronic wounds, owing to their advantageous characteristics. Nevertheless, nanotherapeutics have several obstacles in terms of biological safety, the danger of transdermal toxicities, the variety of persistent wounds, and financial limitations (312, 313). In order to tackle these issues, scholars are now investigating many tactics, including the modification of the physical and chemical characteristics of nanomaterials, the use of stabilizers, and the enhancement of production procedures and nanoformulations. Moreover, a comprehensive comprehension of the metabolic and functional characteristics of animals and humans is essential in the advancement of tailored nanomaterials designed for various wound classifications (314–317). The financial implications associated with nanoformulations provide a significant constraint on the widespread use of nanomaterials in healthcare contexts. The strategies used include dose reduction, incorporation of cost-effective adjuvants, and utilization of microneedles and layered self-assembly techniques to provide regulated and prolonged drug delivery. The decrease in manufacturing costs may be achieved by the optimization of fabrication methods and the development of nanoformulations. The use of nanomaterials in diverse therapeutic formulations and application techniques has been seen, although the underlying processes by which they facilitate wound healing remain inadequately investigated. The TGF-β1/SMAD signaling pathway is well recognized as one of the predominant signaling channels involved in the proliferation phase of wound healing. Additionally, macrophage polarization has been identified as another critical signaling pathway during the inflammatory phase of wound healing (318). There is a need for modeling approaches that are both stimulated and controlled in order to accurately depict the underlying mechanics of wound healing across various kinds of wounds (258).

Stem cell-based treatments have emerged as a prevalent and productive approach to facilitating wound healing. Several tactics, including the investigation of novel stem cell sources, use of stem cell-derived exosomes, and genetic alterations of stem cells, achieve this. Nevertheless, there are still some problems associated with stem cell sources, including genetic instability, immunogenicity, infection risk, carcinogenesis risk, and high processing costs. Autologous iPSC, which are generated from non-viral vectors, are being employed as a strategy to address the challenges posed by immunogenicity and cancer concerns (313, 319). In summary, the potential for enhancing the therapeutic applicability of stem cell-derived stem cells for wound healing may be realized via future developments in GMP-compliant scaling-up technologies (320).

## 6 Conclusions

The maintenance of physiological homeostasis relies heavily on the presence of healthy skin. However, there are several cases when inadequate healing occurs, requiring further intervention. Despite the considerable amount of research conducted and the increased understanding of wound healing procedures, the management of difficult-to-heal chronic wounds and more extensive wounds continues to pose a significant obstacle in the fields of skin regeneration and wound care. Wound healing is a complex process that involves several cellular and molecular systems, hence necessitating the use of multiple treatments rather than relying on a single approach. In order to address these constraints, researchers have investigated a range of novel and developing therapeutic approaches, including nanotherapeutics and stem cell research. These treatments may be used alone or in combination to expedite the process of wound healing, hence offering potential solutions and prospects for managing complicated wounds that are difficult to heal in patients. The challenges pertaining to these unique methodologies and the likely outlook for the future have been deliberated over in order to augment wound care and its administration. In general, the progress made in many technological fields and the discovery of novel and inventive methodologies have significant prospects for enhancing the efficacy of wound healing.

## Author contributions

AM: Writing – original draft, Software, Methodology, Conceptualization, Writing – review & editing, Visualization, Investigation, Formal Analysis. CS: Resources, Project administration, Writing – review & editing, Visualization, Methodology, Investigation, Formal Analysis. PG: Software, Writing – review & editing, Project administration, Methodology, Investigation. SW: Writing – original draft, Visualization, Validation, Supervision, Funding acquisition, Formal Analysis, Writing – review & editing, Investigation. JX: Writing – original draft, Writing – review & editing, Supervision, Investigation, Funding acquisition, Formal Analysis, Conceptualization.
